# Self-Catalyzed Hastelloy Fiber Paper (HAP) Porous Transport Layer for Oxygen Evolution Reaction in Anion Exchange Membrane Water Electrolysis

**DOI:** 10.1021/acs.langmuir.6c04347

**Published:** 2026-10-08

**Authors:** Yu-Chen Chang, Shuo-En Yu, I-Chun Cheng, Jian-Zhang Chen

**Affiliations:** † Graduate School of Advanced Technology, 33561 National Taiwan University, Taipei City 106319, Taiwan; ‡ Institute for Solid State Physics, 13143 The University of Tokyo, Chiba 277-8581, Japan; § Graduate Institute of Photonics and Optoelectronics and Department of Electrical Engineering, National Taiwan University, Taipei City 106319, Taiwan; ∥ Institute of Applied Mechanics, National Taiwan University, Taipei City 106319, Taiwan; ⊥ Advanced Research Center for Green Materials Science and Technology, National Taiwan University, Taipei City 106319, Taiwan; # Research Center for Applied Sciences, Academia Sinica, Taipei City 115201, Taiwan

## Abstract

Hastelloy fiber paper (HAP) can be used as the oxygen evolution reaction porous transport layer (PTL) without using a catalyst layer in anion exchange membrane water electrolysis. It is combined with Ru on nickel fiber paper (NFP) (hydrogen evolution reaction PTL) to form an asymmetric Ru/NFP(−)//HAP­(+) single-cell system. The experimental results for electrodeposited Co­(OH)_2_ on HAP are also demonstrated for comparison. At 70 °C, the cell delivers 725.67 mA cm^–2^ at 1.8 V under cathode-wet conditions and maintains 701.34 mA cm^–2^ under cathode-dry operation. A 5000-cycle accelerated stress test (AST) confirms the good operational stability of the system under dynamic cycling conditions. In addition, the effect of Co­(OH)_2_ electrodeposition on the HAP anode was further investigated to show the current density decreases to 485.59 mA cm^–2^ at 1.8 V when operated at 70 °C. The AST stability also degrades with Co­(OH)_2_ deposition. Further analysis indicates that the electrodeposited Co­(OH)_2_ coating may alter the pore structure and surface wettability of the catalyst layer, leading to mass-transport limitations at high current densities, including hindered ion transport and inefficient oxygen bubble removal.

## Graphical abstract

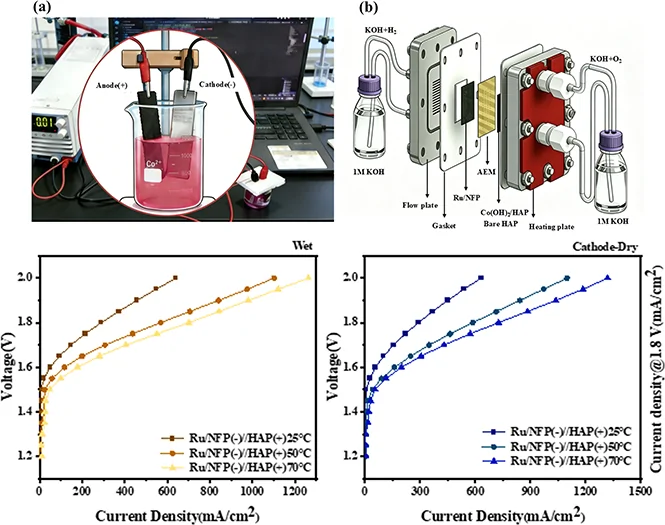

## Introduction

With the continuous increase in global energy demand and the growing impacts of climate change caused by greenhouse gas emissions,^1–3^ the development of low-carbon and even net-zero energy systems has become a central focus of current energy research and policy-making.^4,5^ Among various potential alternative energy carriers and storage solutions, hydrogen, owing to its high gravimetric energy density and the production of only water without direct greenhouse gas emissions during combustion or electrochemical conversion, is widely regarded as a key energy carrier for future energy transitions.^6–8^

However, industrial hydrogen production currently relies predominantly on fossil fuel reforming technologies, such as steam methane reforming, which are highly energy-intensive and associated with substantial carbon dioxide emissions, resulting in so-called “gray hydrogen” that fundamentally undermines hydrogen’s environmental advantage as a low-carbon energy carrier and limits its sustainability under long-term deep decarbonization scenarios.^9,10^ The International Energy Agency (IEA) has repeatedly emphasized the urgency of phasing out fossil fuel dependence and has explicitly called for all countries to achieve a fully decarbonized energy and societal system by 2050 to meet global climate mitigation goals and temperature control pathways.^11–13^ Guided by this vision, an increasing number of countries have incorporated net-zero and deep decarbonization targets into their national energy and industrial policy frameworks, actively promoting the large-scale deployment of hydrogen technologies and renewable energy, and positioning “green hydrogen” as a cornerstone for future zero-carbon energy systems.^14,15^

Producing hydrogen via water electrolysis powered by renewable electricity is widely recognized as a critical technological pathway for achieving a low-carbon or even net-zero-carbon hydrogen supply. Water electrolysis not only avoids direct fossil fuel utilization but also serves as a means of converting and storing renewable energy, transforming intermittent electricity into chemical energy that can be stored in the long term and utilized across sectors, thereby playing a pivotal role in balancing and supporting future energy systems with high shares of renewable energy.^16–18^

With the rapid development of renewable energy technologies, producing hydrogen via water electrolysis powered by renewable electricity is considered to be an effective pathway for green hydrogen production. Traditional alkaline water electrolysis (AWE) offers advantages such as a mature system design, operational stability, and flexibility in material selection; however, its liquid electrolyte configuration limits the achievable current density and dynamic operational capability.^19^ In contrast, proton-exchange-membrane water electrolysis (PEMWE) can operate at high current densities and generate high-purity hydrogen, but its requirement for strongly acidic conditions necessitates a heavy reliance on noble-metal catalysts and corrosion-resistant structural materials, consequently increasing system costs.^20,21^

To combine the material flexibility achievable under alkaline operating conditions with the high-performance characteristics of solid-state electrolyzer systems, anion exchange membrane water electrolysis (AEMWE) has attracted considerable attention in recent years.^22–25^ AEMWE employs an anion exchange membrane to conduct hydroxide ions, enabling electrode reactions to occur under alkaline conditions while retaining the high-current-density operational potential afforded by zero-gap configurations.^26^ However, this technology still faces challenges, including insufficient membrane chemical stability, limited ionic conductivity, and high electrode–membrane interfacial resistance. These issues are particularly pronounced under pure-water operation, where they can significantly impact the overall system performance.^27,28^

The oxygen evolution reaction (OER) is widely recognized as the kinetically most sluggish half-reaction in water electrolysis,^29–31^ as its overpotential directly governs the operating cell voltage and overall energy efficiency, particularly under high-current density conditions where energy losses become increasingly significant.^32^ In alkaline environments, cobalt and its corresponding oxides or hydroxides (e.g., CoOOH and Co_3_O_4_) have been extensively reported to exhibit outstanding OER catalytic activity, enabling substantial overpotential reduction without reliance on scarce and expensive noble metals such as iridium (Ir) or ruthenium (Ru).^33^ In addition to their high intrinsic activity, Co-based materials demonstrate favorable electrochemical stability, corrosion resistance, and long-term durability, rendering them among the most promising nonprecious metal OER catalysts for alkaline water electrolysis. The effectiveness of cobalt primarily originates from its ability to form highly active oxide or oxyhydroxide phases (e.g., CoOOH) under alkaline conditions, which exhibit strong adsorption toward water molecules and hydroxide ions, thereby facilitating charge transfer and accelerating the OER reaction kinetics.^34^

On the basis of these considerations, Hastelloy fiber paper (HAP) was selected as the anode substrate in this study. As a high-nickel-content corrosion-resistant alloy, Hastelloy offers excellent chemical stability, superior resistance to strong alkaline corrosion, and high mechanical strength, effectively suppressing metal dissolution and structural degradation during electrochemical operation.^35^ These properties ensure stable electrochemical performance and structural integrity under prolonged operation and high-current-density conditions. Moreover, Hastelloy exhibits high-temperature tolerance, resistance to stress corrosion cracking, and exceptional toughness, making it particularly suitable for industrial-scale alkaline-water electrolysis systems.

In this study, HAP is demonstrated to exhibit intrinsic OER activity without the intentional deposition of an additional catalyst layer. This intrinsic catalytic behavior enables HAP to simultaneously serve as both the porous transport layer and the OER-active electrode, which is herein referred to as a “self-catalyzed” PTL. Unlike conventional PTLs that primarily provide mass transport and electrical conduction, the HAP investigated in this work contributes directly to the OER reaction. The catalytic behavior originates from the alloy surface itself, which contains catalytically relevant transition-metal species including Ni, Mo, and Cr. Under alkaline anodic conditions, the surface may undergo oxidation and reconstruction, generating electrochemically active surface species. Although the individual contributions of Ni, Mo, and Cr cannot be completely separated based on the present experiments, their combined surface chemistry is considered responsible for the intrinsic OER activity of HAP. The performance of the Co­(OH)_2_ layer electrodeposited onto HAP PTL is also compared. Both HAP and Co­(OH)_2_/HAP are tested as the anode for AMEWE with Ru hydrothermally grown on nickel fiber paper (NFP) as the cathode.

## Experiments

### PTL Pretreatment

NFP and HAP were employed as porous substrates in this study. Both materials were purchased from Koolon Advanced Material Technology Co. Ltd. (Taiwan). The NFP had a thickness of 0.25–0.30 mm, a fiber diameter of 12 μm, and a porosity of 56%, whereas the HAP had a thickness of 0.47 mm, a fiber diameter of 20 μm, and a porosity of 82%.

The materials were cut into 5 cm × 5 cm specimens and sequentially immersed in 1 M sulfuric acid, ethanol, and deionized water, with ultrasonication for 15 min in each solution, to remove native oxide layers and surface impurities. Subsequently, the samples were dried in an oven at 70 °C for 2 h.

### Preparation of Co­(OH)_2_/HAP

An electroplating solution was prepared by dissolving 1 g of Co­(NO_3_)_2_·6H_2_O in 150 mL of deionized water. The pretreated HAP was immersed in the electroplating solution, with carbon fiber paper (GPP035, CeTech Co. Ltd.) with a thickness of 0.35 mm serving as the anode and HAP as the cathode. The electrodeposition was carried out over an exposed area of 3 cm × 3 cm. The mass loading of the electrodeposited Co­(OH)_2_ on HAP was determined to be 1.2 mg cm^–2^, calculated from the weight difference before and after deposition. Pulsed current electrodeposition was performed using alternating low and high currents of 0.09 and 2.0 A, respectively, each applied for 1 s, for a total of 300 cycles. The pulse-electrodeposition parameters were selected based on preliminary deposition trials. The low-current stage (0.09 A) was employed to promote controlled nucleation and coating uniformity on the porous HAP substrate, whereas the high-current stage (2.0 A) was used to increase the deposition rate and achieve sufficient cobalt loading within a practical processing time. The total number of 300 cycles was selected to obtain continuous cobalt coverage while maintaining the porous fibrous structure of HAP. After electroplating, the samples were dried in an oven at 70 °C for 1 h.

### Hydrothermal Synthesis of Ru/NFP Cathode

5 mmol of RuCl_3_·3H_2_O (1.307 g) was dissolved in a solvent mixture of 80 mL ethylene glycol and 80 mL deionized water. The solution was magnetically stirred for 1 h to ensure complete dissolution. The cleaned NFP substrates were then immersed in the prepared solution and transferred into a Teflon-lined stainless-steel autoclave. The autoclave was sealed and heated at 160 °C for 16 h in an oven to facilitate the hydrothermal deposition of Ru on the NFP substrate. After naturally cooling to room temperature, the obtained sample was thoroughly rinsed with deionized water and dried in an oven for 2 h.

### Material Characterizations

To investigate the electroplated materials and the morphological evolution of the surface before and after long-term AMEWE operation, multiple characterization techniques were employed. X-ray diffraction (XRD; Bruker D2 PHASER) was used to analyze the crystalline structure, with a 2θ scanning range of 5°–70°. Surface morphology was characterized using a scanning electron microscope (SEM; JEOL JSM-6500F), and elemental distribution was analyzed by an energy-dispersive X-ray spectrometer (EDS; Oxford Instruments) attached to the SEM, with images acquired at a magnification of 5000×. X-ray photoelectron spectroscopy (XPS; Sigma Probe, Thermo VG Scientific) equipped with an Al Kα radiation source was employed to investigate surface chemical bonding states, oxidation states, and elemental composition. Water contact angles were measured using a contact angle goniometer (Model 100SB, Sindatek) to evaluate the surface hydrophilicity.

### Electrochemical Measurements

All electrochemical measurements were carried out using an Autolab PGSTAT204 electrochemical workstation (Metrohm, Netherlands) in 1 M KOH electrolyte at room temperature. A standard three-electrode configuration was employed. The samples were cut into dimensions of 3 cm × 1 cm, with an area of 2 cm × 1 cm immersed in the electrolyte and used as the working electrode. A platinum electrode and an Ag/AgCl electrode were used as the counter electrode and reference electrode, respectively. The measured potentials versus Ag/AgCl were converted to the reversible hydrogen electrode (RHE) scale using the Nernst eq 1

1 $$E_{\mathrm{RHE}}=E_{\mathrm{Ag}/\mathrm{AgCl}}+0.059\times \mathrm{pH}+0.197$$

Linear sweep voltammetry was performed at a scan rate of 5 mV s^–1^ to get the polarization curves; 85% *iR* compensation was applied to minimize the influence of solution resistance on the electrochemical measurements. The *iR*-corrected potential was calculated according to eq ([Disp-formula eq2])

2 $$E_{\mathrm{corrected}}=E_{\mathrm{measured}}-0.85\times I\times R_{\mathrm{solution}}$$

Electrochemical impedance spectroscopy measurements were conducted over a frequency range of 0.1 Hz to 10 kHz at an overpotential of 400 mV to evaluate the charge-transfer resistance (*R*_ct_). Cyclic voltammetry measurements were performed in the potential range from −0.05 V to −0.15 V versus Ag/AgCl at various scan rates of 20, 50, 100, 150, 200, 250, and 300 mV s^–1^ to determine the double-layer capacitance (2*C*_dl_).

### AEMWE Module

The commercial AEMWE module used in this study was purchased from Dioxide Materials. The module is based on nickel flow plates with a single serpentine flow channel, offering an active electrocatalytic area of ∼5 cm^2^. The cathode, prepared via a hydrothermal method, consists of a Ru/NFP electrode for the hydrogen evolution reaction (HER), while the anode and cathode are separated by an anion exchange membrane (Fumasep FAA-3–50), which was presoaked in 1 M KOH for 24 h prior to assembly. The electrolyte is 1 M KOH, circulated through the anode and cathode at a flow rate of 10 mL/min by using a peristaltic pump to ensure continuous flow and stable reaction conditions.

Thermal management of the module is achieved using a proportional-integral-derivative (PID) controller (DTA4848 V1, Delta Electronics, Taipei, Taiwan), which regulates a 100 W heating plate in direct contact with the nickel flow plates. The flow plate temperature was continuously monitored using a K-type thermocouple to maintain stable operating conditions. Electrical power is supplied by a regulated power supply (SPS-1230, GWInstek, New Taipei City, Taiwan) capable of delivering both continuous and pulsed currents. The actual operating voltage of the module was continuously monitored using a digital multimeter (15B, FLUKE, Everett, WA, USA). To ensure safe operation and protect the module, the applied voltage was strictly limited to below 2 V. All polarization curve measurements were conducted without an *iR* compensation.

Electrochemical evaluation was performed in multiple stages to assess catalytic performance and stability. Initially, polarization curves were recorded at 25 °C, 50 °C, and 70 °C to investigate the electrochemical behavior of the cathode under both dry (cathode-dry) and wet (cathode-wet) operating modes. Subsequently, to verify the structural stability of the electrodes under dynamic loading, accelerated stress tests (AST) were conducted at 25 °C under cathode-wet conditions. The AST protocol comprised 5,000 cycles of alternating high and low current densities (500/100 mA cm^–2^) with a switching frequency of 0.1 Hz.

The AST parameters were selected to impose repeated current density fluctuations representative of dynamic operating conditions. Alternating between 100 and 500 mA cm^–2^ allows the electrodes to experience both moderate and relatively high operating loads, while the 5 s interval (0.1 Hz) generates frequent transitions that accelerate surface reconstruction and degradation processes within a practical experimental duration. Overall, these findings highlight the importance of balancing intrinsic catalytic activity with optimized mass transport in the AEMWE. Finally, a short-term galvanostatic stability test was performed at a constant current density of 500 mA cm^–2^ for 60 h. This stage specifically incorporated an intermittent startup-shutdown protocol every 12 h, aimed at comprehensively evaluating the catalyst’s ability to maintain activity under extreme cyclic operating environments.

## Results and Discussion

### Materials and AEMWE Overall Architecture

As illustrated in Figure 1(a), the Co­(OH)_2_ was directly and in situ electrodeposited onto the acid-etched HAP substrate, resulting in a binder-free, monolithic, and fully integrated electrode architecture. Such an integrated electrode configuration effectively eliminates the interfacial resistance between the catalyst layer (CL) and the porous transport layer (PTL), thereby facilitating more efficient electron transport and accelerating interfacial charge-transfer kinetics.

**Figure 1:**
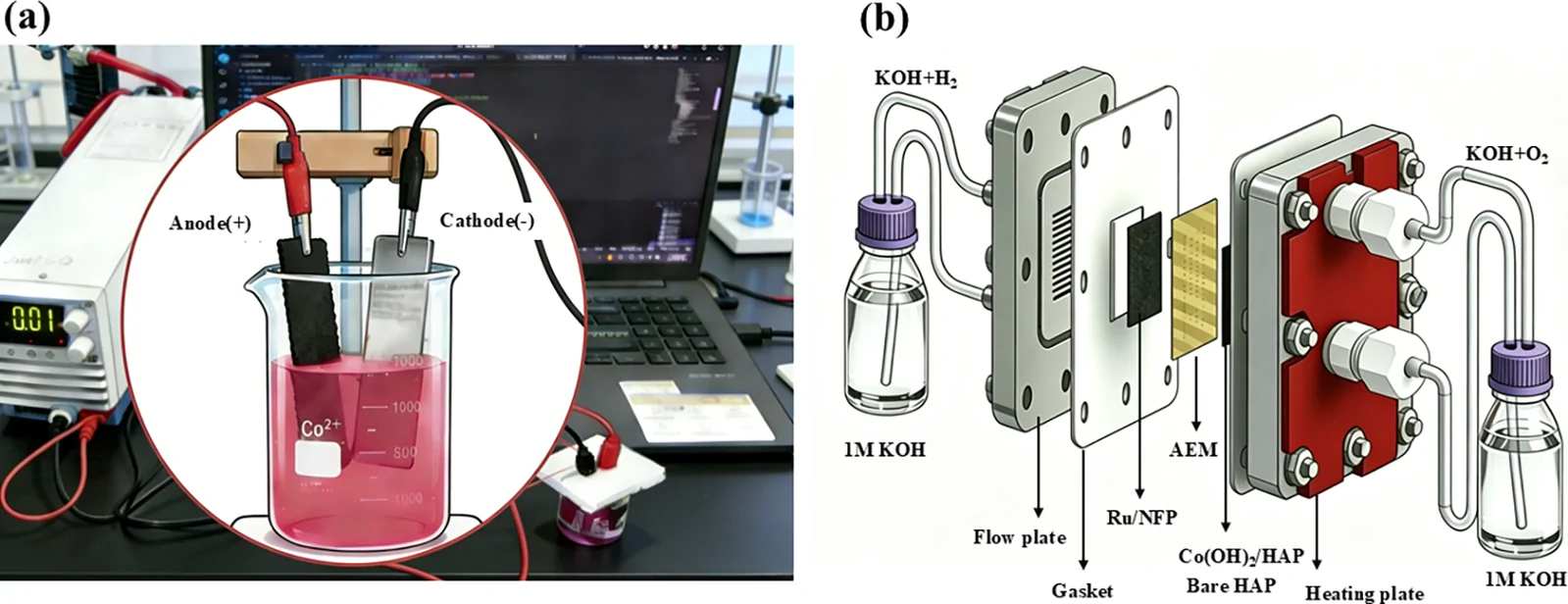
Schematic diagrams of (a) the assembled AEMWE single cell and (b) the electrode fabrication setup via electroplating.

Figure 1(b) Configuration of the AEMWE single cell assembled with a Co­(OH)_2_/HAP anode and a Ru/NFP cathode. To assess the electrocatalytic performance of the catalyst layer (CL)/porous transport layer (PTL) interface under practical operating conditions, we evaluated both pristine HAP and Co­(OH)_2_/HAP electrodes.

### Material Characterization

As shown in Figure 2, all samples exhibited favorable hydrophilic characteristics. Upon contact with the electrode surfaces, the water droplets were rapidly absorbed into the porous structure, indicating a favorable surface wetting and liquid infiltration. After Co­(OH)_2_ electrodeposition, the electrodes maintained a similar rapid water absorption behavior, suggesting that the electrodeposition process did not substantially alter the apparent wettability of the HAP-based electrodes. This favorable wetting behavior is consistent with an effective liquid infiltration into the porous electrode structure. However, the similar wetting behavior before and after Co­(OH)_2_ deposition suggests that the lower single-cell performance of Co­(OH)_2_/HAP cannot be attributed solely to changes in the surface wettability. Other factors, including catalyst loading and structural or mass-transport effects within the porous electrode, may also contribute to the observed performance difference.

**Figure 2:**
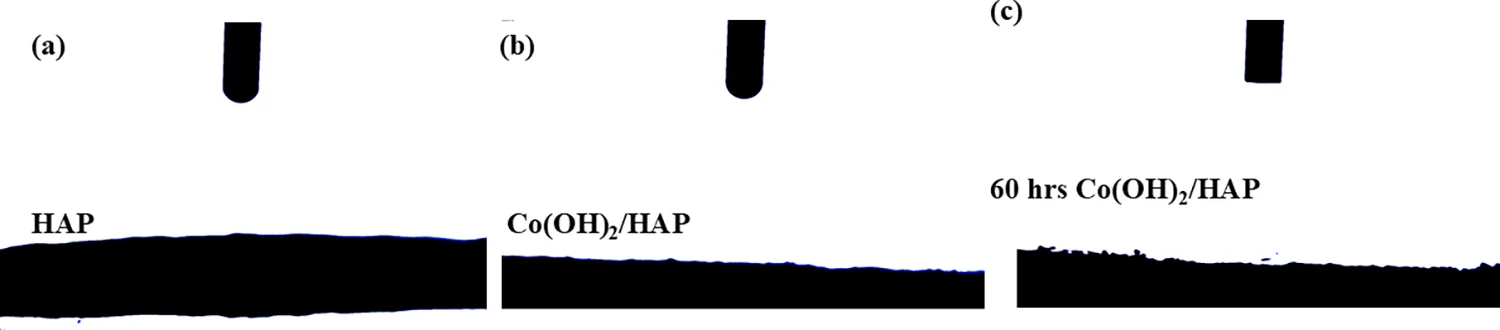
Water contact angles for (a) HAP, (b) Co­(OH)_2_/HAP, (c) 60 h Co­(OH)_2_/HAP.

As shown in Figure 3, the characteristic diffraction peaks of HAP at the (111) and (200) planes confirm the well-crystallized face-centered cubic structure of the substrate. After accelerated stress testing (AST) and prolonged operation, the (111) peak remains prominent and stable, indicating that the metallic framework of HAP maintains excellent structural integrity under alkaline conditions.

**Figure 3:**
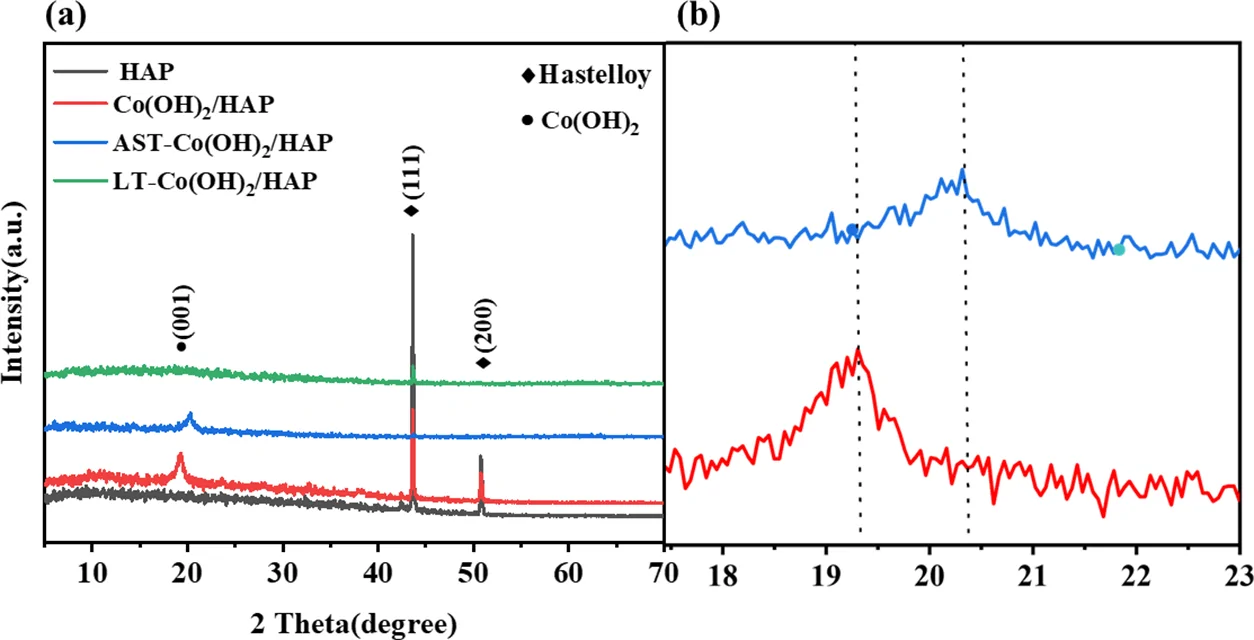
(a) XRD patterns of HAP, Co­(OH)_2_/HAP, AST-Co­(OH)_2_/HAP, and 60 h-Co­(OH)_2_/HAP; (b) enlarged view of the diffraction peak at 18–23°.

For the Co electrodeposited samples, a diffraction peak corresponding to the Co­(OH)_2_(001) plane is initially observed. Notably, a discernible peak shift occurs following AST, which can be primarily attributed to the in situ surface reconstruction of the catalyst, transforming the initial hydroxide precursor Co­(OH)_2_ into the electrochemically active oxyhydroxide phase CoOOH.^36,37^ This structural evolution often induces lattice strain and local coordination changes, accounting for the observed shift in the diffraction angles. Furthermore, the peak intensity significantly decreases after long-term operation and eventually becomes barely detectable.

This trend indicates a reduction in long-range crystallinity and may be associated with both partial cobalt depletion and transformation of the remaining cobalt species into poorly crystalline, nanocrystalline, or amorphous phases. Because such phases exhibit limited long-range order, they may not be readily detected by XRD despite remaining electrochemically relevant under OER conditions.^38^ This interpretation is also consistent with the SEM and XPS results, which show residual cobalt-related surface features and detectable Co signals after 60 h of operation, although at significantly reduced intensity.^38^

Figure 4 illustrates the morphological evolution of the HAP-based electrodes before and after Co electrodeposition as well as following durability tests. The pristine HAP (Figure 4a) exhibits a well-defined fibrous architecture with a smooth surface. After electrodeposition (Figure 4b), cobalt species are uniformly deposited onto the HAP framework, while largely preserving the original fibrous structure. After 5000 potential cycles (AST, Figure 4c), significant structural reconstruction is observed. The initially well-dispersed cobalt species aggregate into spherical or blocklike domains, and the fibrous features become less distinct, indicating pronounced morphological change under repeated high anodic polarization. In contrast, after 60 h of continuous OER operation (Figure 4d), the structural evolution is more gradual.

**Figure 4:**
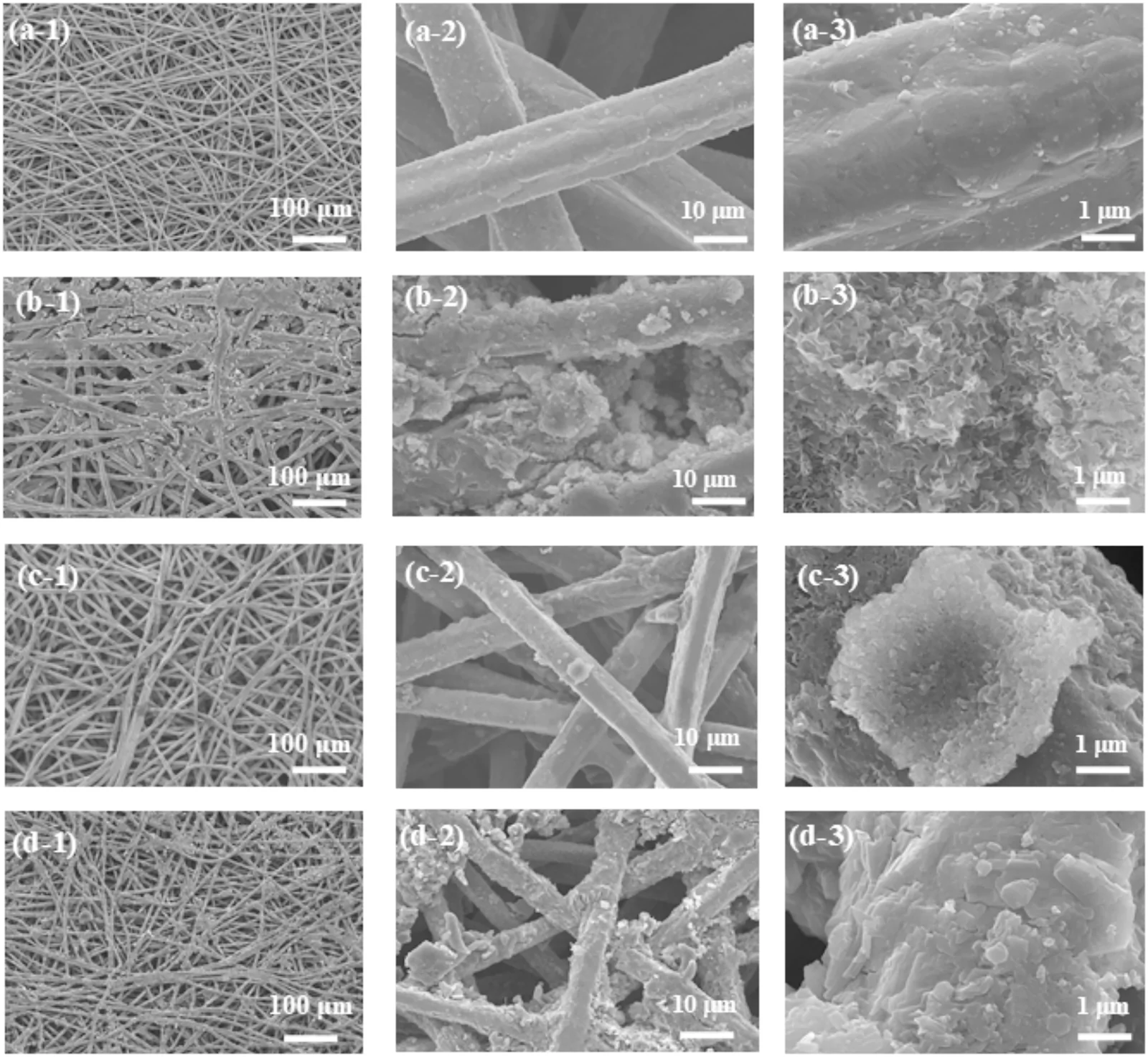
SEM micrographs of (a) HAP, (b) Co­(OH)_2_/HAP, (c) AST-treated Co­(OH)_2_/HAP, and (d) 60 h-treated Co­(OH)_2_/HAP at magnifications of 100×, 1000×, and 10,000×.

Although cobalt aggregation remains evident, the fibrous framework is still partially retained. This suggests that sustained operation induces slower surface restructuring than aggressive potential cycling, while prolonged electrolysis nevertheless promotes cobalt coalescence and a gradual reduction in the active surface area.^39^ The SEM observation provides qualitative evidence of morphology evolution, but it does not directly quantify surface roughness or porosity changes.

To further investigate the surface elemental composition and the chemical evolution of the electrodes under different conditions, XPS survey spectra were recorded and analyzed, as presented in Figure 5. The survey spectrum of the pristine HAP sample clearly exhibits characteristic signals of Ni 2p, Mo 3d, and Cr 2p, which originate from the alloy substrate, thereby confirming the inherent composition of the underlying Hastelloy framework.

**Figure 5:**
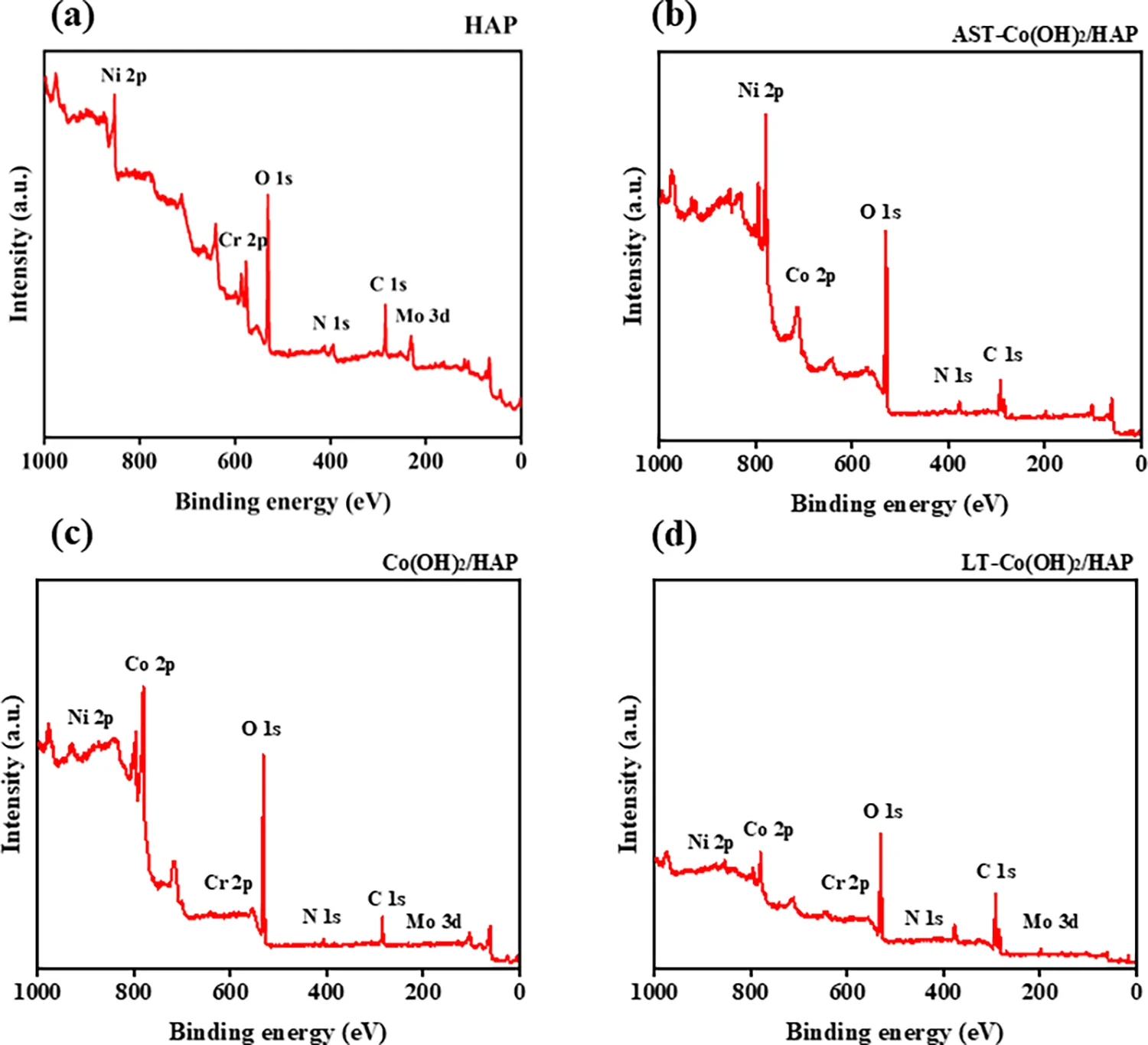
XPS survey spectra of (a) HAP, (b) Co­(OH)_2_/HAP, (c) AST-treated Co­(OH)_2_/HAP, and (d) 60 h-treated Co­(OH)_2_/HAP.

Following the Co electrodeposition, a distinct and intense Co 2p signal emerges in the Co/HAP sample, providing direct evidence for the successful loading of cobalt-based catalytic species onto the HAP surface. Concurrently, while the Ni signal remains detectable, the intensities of Mo and Cr signals undergo a significant reduction, with the Cr signal becoming nearly indistinguishable from the background noise. This observation suggests that the electrodeposited cobalt layer effectively encapsulates the substrate, exceeding the typical XPS escape depth (approximately 5–10 nm) and shielding the signals from the underlying Mo and Cr species.

The surface stability was further evaluated after the electrochemical treatments. Interestingly, for the AST-treated sample, although SEM observations (Figure 4c) indicate a substantial decrease in visible cobalt coverage and a change in surface morphology, the Co 2p signal remains remarkably prominent in the XPS spectra. This discrepancy implies that the cobalt species are not entirely stripped from the electrode; instead, they likely undergo a surface reconstruction and redistribution process, transitioning from a uniform coating into localized, perhaps more chemically stable clusters or oxyhydroxide phases during the accelerated potential cycling.^40,41^

In contrast, the 60 h-treated sample exhibits a markedly weakened Co signal alongside a resurgence of the substrate signals (Ni, Mo, and Cr). This indicates more severe and irreversible cobalt depletion after prolonged operation. Combined with the SEM results, these findings suggest a distinct degradation mechanism: while AST primarily induces morphological restructuring and localized concentration of active sites, long-term operation under sustained oxygen evolution conditions leads to the gradual dissolution or mechanical detachment of the catalyst layer. Such depletion is likely attributed to the limited interfacial adhesion between the electrodeposited cobalt and the HAP substrate, where the continuous evolution of O_2_ bubbles at the interface exerts mechanical stress, eventually leading to the loss of active material and a decrease in electrochemical durability.^42,43^

To investigate the elemental composition and chemical states on the material surface, high-resolution (HR) XPS analysis was performed in Figure 6. The results indicate that all samples exhibit distinct characteristic signals. HR spectra of the Ni 2p region can be deconvoluted into multiple spin–orbit doublets. Specifically, Ni^2+^ is assigned to the peaks at 2p_3/2_ (∼855.7 eV) and 2p_1/2_ (∼873.6 eV),^44,45^ while Ni^3+^ corresponds to 2p_3/2_ (∼856.8 eV) and 2p_1/2_ (∼875.1 eV).^46^ In addition, metallic Ni^0^ signals are observed at 852.9 and 870.2 eV,^47^ along with characteristic shakeup satellite peaks in the range of 861–864 eV.

**Figure 6:**
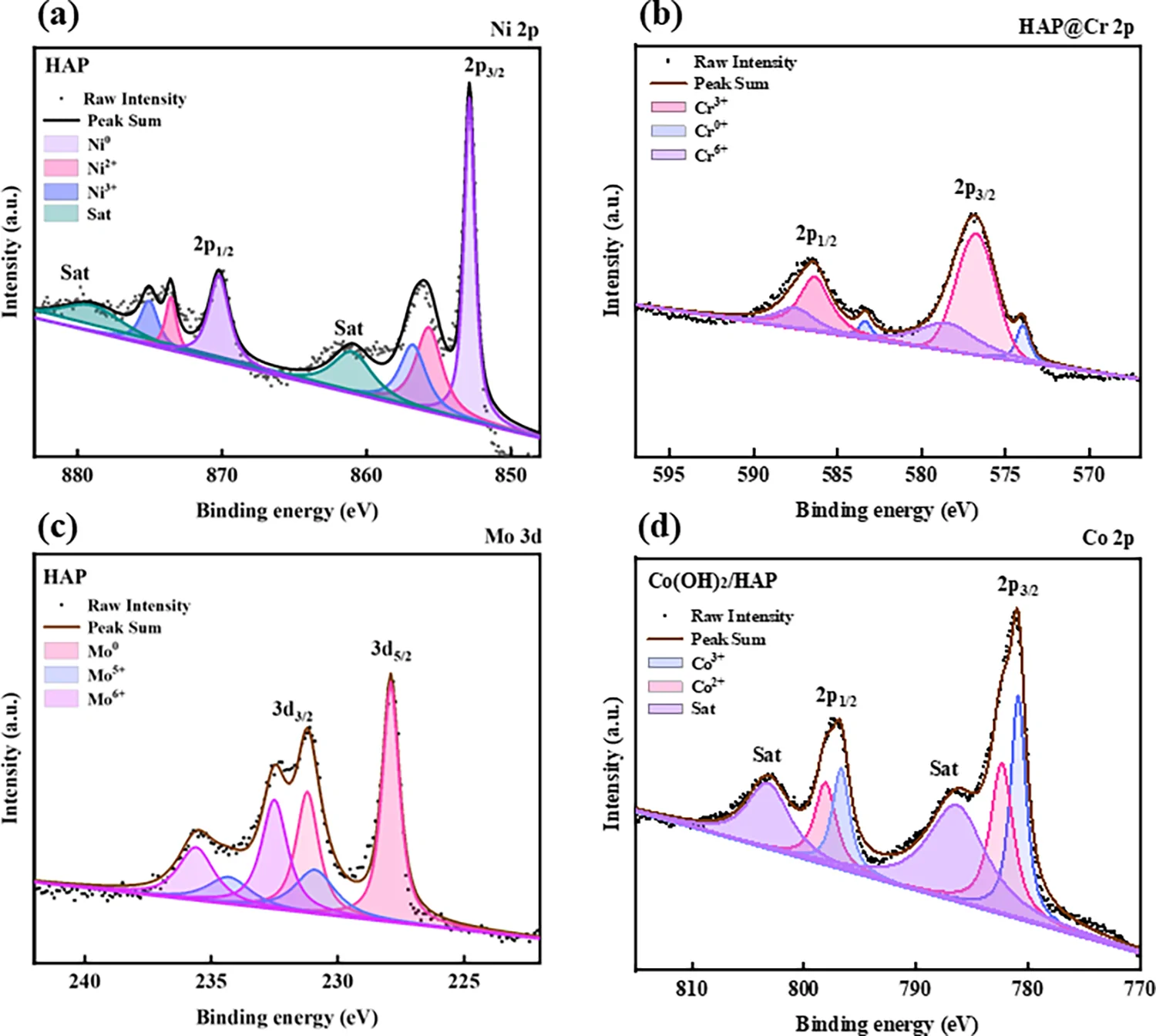
HR XPS spectra of (a) Ni 2p, (b) Cr 2p, and (c) Mo 3d for the pristine HAP substrate, and (d) Co 2p for the Co­(OH)_2_/HAP electrode.

The HR Co 2p spectra reveal that Co^3+^ is located at 2p_3/2_ (780.9 eV) and 2p_1/2_ (796.6 eV), whereas Co^2+^ is assigned to 782.4 and 798.1 eV,^48–50^ accompanied by distinct satellite peaks at approximately 786 and 803 eV. The Cr 2p spectra demonstrate the coexistence of metallic Cr^0^ (2p_3/2_, 573.97 eV; 2p_1/2_, 583.35 eV), Cr^3+^ (2p_3/2_, 576.77 eV; 2p_1/2_, 586.36 eV), and Cr^6+^ (2p_3/2_, 578.9 eV; 2p_1/2_, 587.6 eV), indicating the presence of multiple oxidation states on the electrode surface.^51,52^ Furthermore, the Mo 3d region can be deconvoluted into three sets of spin–orbit doublets, including Mo^6+^ (3d_5/2_ 232.5 eV, 3d_3/2_ 235.6 eV), Mo^5+^ (3d_5/2_ 230.9 eV, 3d_3/2_ 234.3 eV), and Mo^0^ (3d_5/2_ 227.9 eV, 3d_3_ 231.2 eV), suggesting that partial reduction occurred during the synthesis process.^53,54^

The results further support the electrochemical activity of the HAP surface. The pristine HAP exhibits Ni, Cr, and Mo species with multiple oxidation states, indicating that the alloy surface contains chemically active transition-metal species rather than behaving as an electrochemically inert structural support. In combination with the OER polarization results obtained from pristine HAP, these observations support the intrinsic catalytic nature of the HAP surface. Therefore, HAP functions not only as a porous transport medium but also as an OER-active electrode without an intentionally deposited catalyst layer, supporting its description as a self-catalyzed PTL.

As illustrated by the HR Co 2p spectra in Figure 7, the proportion of Co^3+^ species significantly increased from 42.31% to 47.26% after the AST treatment, accompanied by a corresponding decrease in the Co^2+^ fraction. This evolution in the electronic structure provides strong evidence for the in situ surface reconstruction of the catalyst from Co­(OH)_2_ to CoOOH.^55,56^ According to previous studies, CoOOH is widely recognized as the actual active phase for the alkaline oxygen evolution reaction (OER), rather than the initial hydroxide precursor.^57^ The electrochemically induced formation of CoOOH leads to a more favorable electronic configuration, which effectively regulates the adsorption strength of key reaction intermediates (e.g., OH* and O*). This modulation lowers the activation energy barrier and enhances charge-transfer kinetics.^58^ Therefore, the conversion from Co^2+^ to Co^3+^ during the AST process not only increases the density of active sites but also optimizes reaction kinetics. This transformation plays a crucial role in improving the long-term catalytic activity and stability.

**Figure 7:**
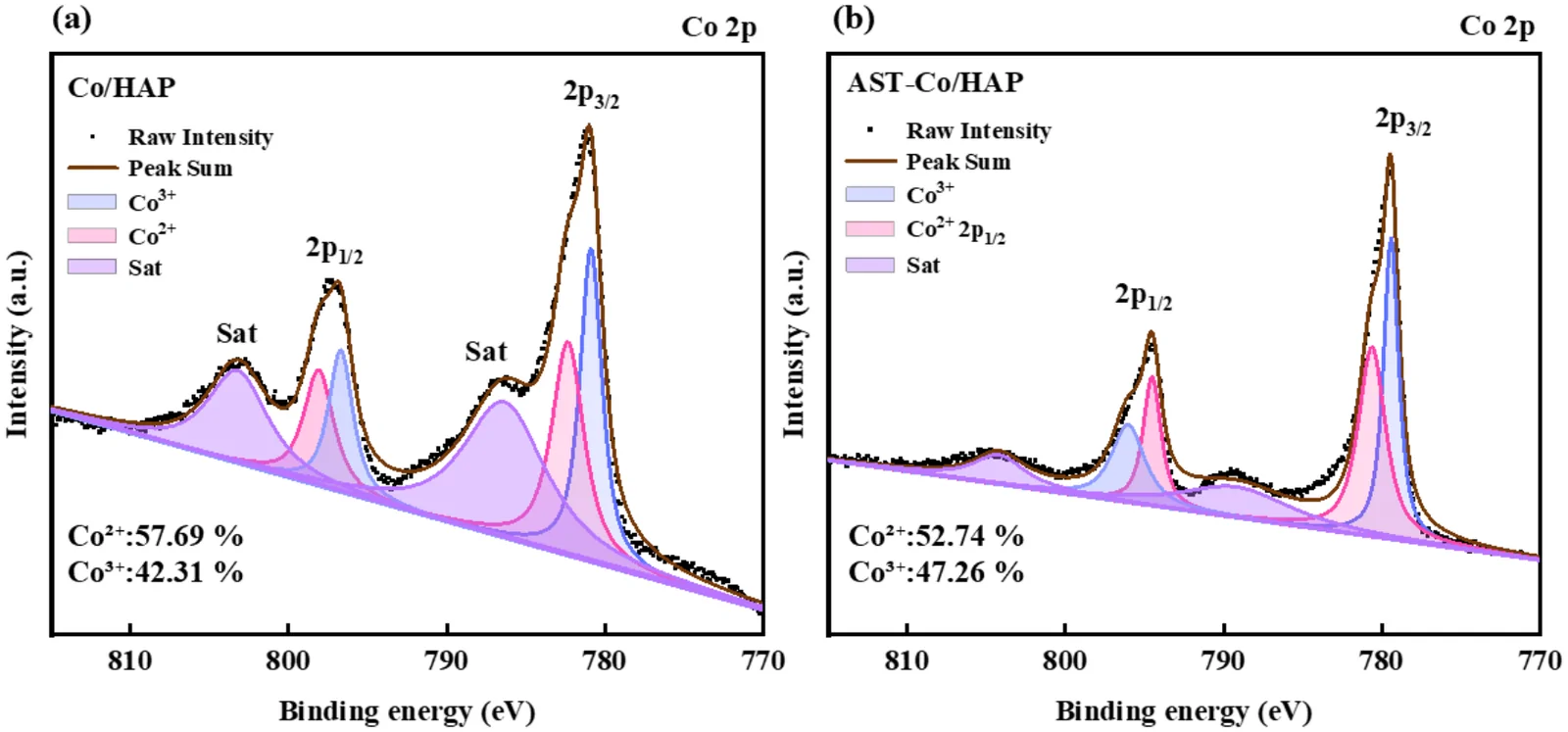
HR XPS spectra of Co 2p for (a) Co­(OH)_2_-HAP and (b) AST Co­(OH)_2_-HAP samples.

### Electrochemical Analysis

As shown in Figure 8, after the deposition of a Co metallic thin film by electrodeposition, the Co­(OH)_2_/HAP electrode exhibited slightly better oxygen evolution reaction (OER) catalytic performance compared with the pristine HAP substrate in 1.0 M KOH electrolyte. According to the LSV polarization curves in Figure 8(a) and 8(c), the overpotentials required for the pristine HAP at current densities of 10, 50, and 100 mA cm^–2^ were 363, 556, and 757 mV, respectively, whereas those of Co­(OH)_2_/HAP decreased to 341, 536, and 744 mV, respectively. These results indicate that the deposition of the Co­(OH)_2_ thin film reduces the energy barrier required for the reaction and enhances the OER catalytic efficiency of the electrode. In particular, the decrease in overpotential observed at all current density conditions further confirms the positive effect of the Co­(OH)_2_ on OER activity.^59^ Although the improvement in OER catalytic performance upon Co­(OH)_2_ deposition is modest, we still performed further analyses incorporating additional electrochemical results.

**Figure 8:**
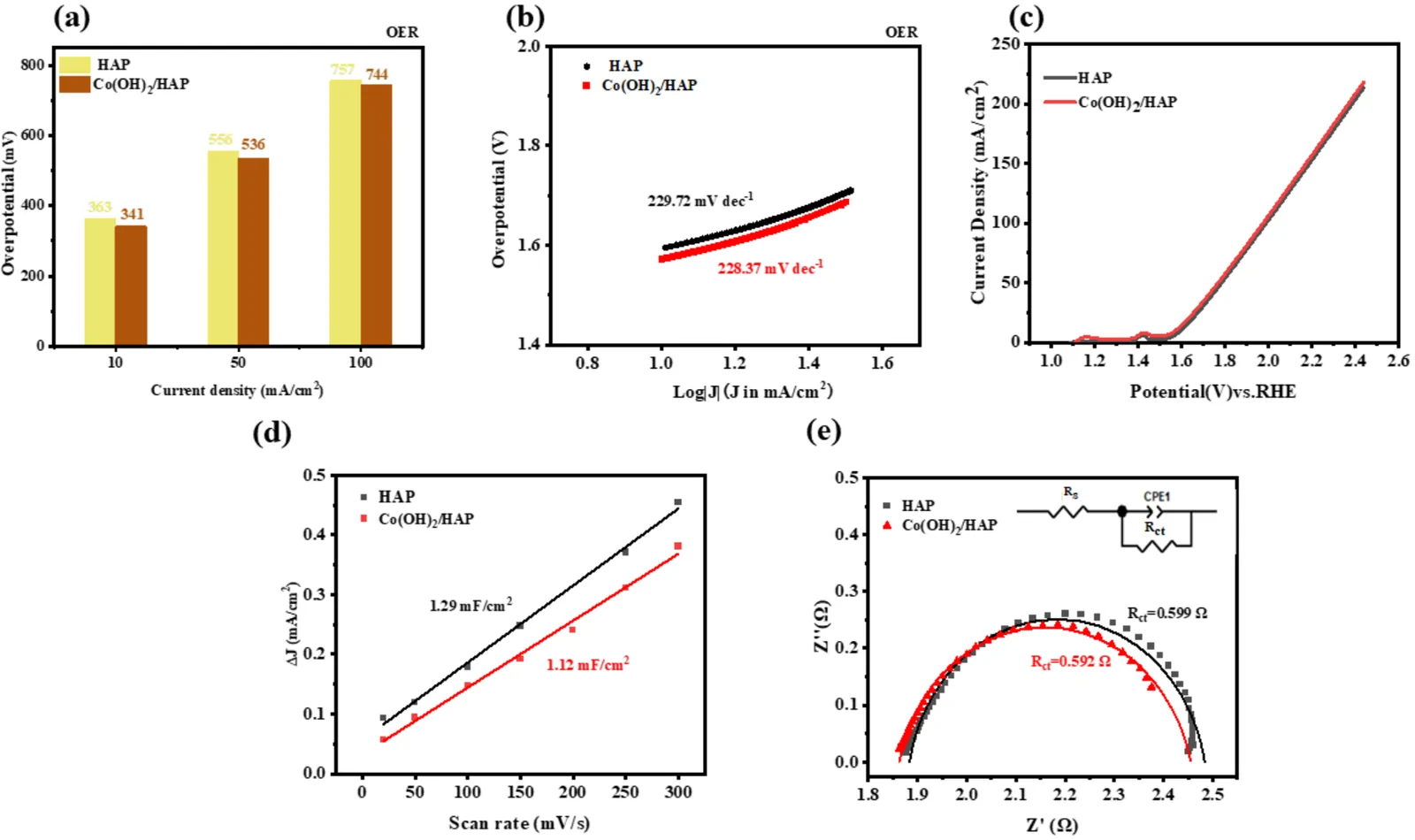
Electrochemical OER performance of HAP and Co­(OH)_2_/HAP electrodes: (a) overpotentials at 10, 50, and 100 mA cm^–2^, (b) Tafel plots, (c) LSV polarization curves, (d) double-layer capacitance measurements, and (e) Nyquist plots with the corresponding equivalent circuit model.

To further investigate the reaction kinetics, the Tafel plots shown in Figure 8(b) reveal that the Tafel slopes of HAP and Co­(OH)_2_/HAP are 229.72 and 228.37 mV dec^–1^, respectively, which are very close to each other. The nearly identical Tafel slopes suggest that Co­(OH)_2_ deposition does not substantially alter the apparent OER reaction pathway or rate-determining behavior under the tested conditions.

The relatively high Tafel slopes indicate that the overall OER process remains subject to considerable apparent kinetic limitations. In a three-electrode configuration using porous fibrous electrodes, the measured Tafel slopes may reflect not only intrinsic OER charge-transfer kinetics but also contributions from local OH^–^ transport, oxygen bubble accumulation, and mass-transport effects within the porous structure. Therefore, the measured Tafel slopes should be regarded as apparent kinetic parameters rather than as direct evidence of a specific elementary OER mechanism. Although Co­(OH)_2_/HAP exhibits lower overpotentials than pristine HAP, the comparable Tafel slopes suggest that the performance improvement is primarily reflected by the reduced overpotential rather than a substantial change in the apparent OER mechanism.^60^

In addition, to evaluate the effective active surface area of the electrode, the double-layer capacitance (*C*_dl_) was obtained from the cyclic voltammetry (CV) measurements shown in Figure 8(d), which was further used to estimate the electrochemically active surface area (ECSA). The results show that the 2*C*_dl_ value of pristine HAP is 1.29 mF cm^–2^, while that of Co­(OH)_2_/HAP is 1.12 mF cm^–2^. Although the 2*C*_dl_ value of Co­(OH)_2_/HAP is slightly lower, indicating that its apparent active surface area did not increase and even slightly decreased, its OER performance was still significantly improved. This suggests that the enhancement in performance is not primarily attributed to an increase in the number of active sites but is more likely related to the higher intrinsic catalytic activity of the Co­(OH)_2_ thin film itself.^61^

This interpretation is further supported by the electrochemical impedance spectroscopy (EIS) results shown in Figure 8(e). The charge-transfer resistance (*R*_ct_) of pristine HAP was 0.599 Ω, which slightly decreased to 0.592 Ω after Co­(OH)_2_ deposition. The observed difference in *R*_ct_ is relatively small, suggesting that the charge-transfer characteristics of HAP and Co­(OH)_2_/HAP are largely comparable under the tested conditions. Nevertheless, the slightly lower *R*_ct_ value of Co­(OH)_2_/HAP may indicate a modest improvement in interfacial charge transfer.

Taken together, the LSV, Tafel, CV, and EIS analyses suggest that the improved OER activity of the Co­(OH)_2_/HAP electrode is primarily associated with the intrinsic activity of the Co-based surface species, while only minor differences in the charge-transfer resistance were observed between the two electrodes. Therefore, the enhanced OER performance cannot be solely attributed to a reduction in *R*_ct_ but is more likely related to the intrinsic electrocatalytic characteristics of the Co-containing surface layer.^62^

### AEMWE Performance Evaluation

Figure 9 presents the polarization behavior and durability performance of single-cell systems Ru/NFP(−)//HAP­(+) and Ru/NFP(−)//Co­(OH)_2_/HAP­(+) under different operating conditions. As shown in Figures 9(a,b,e,f), at 25, 50, and 70 °C, the current density of both systems increases with temperature regardless of whether the cathode is under dry or wet conditions. Moreover, the performance under wet conditions is significantly higher than that under dry conditions, indicating that proper membrane hydration and high-temperature operation facilitate OH^–^ transport and reaction kinetics. In addition, as illustrated in Figure 9(c,g), at a constant voltage of 1.8 V, wet conditions and high-temperature operation also contribute significantly to the enhancement of current density.^63^

**Figure 9:**
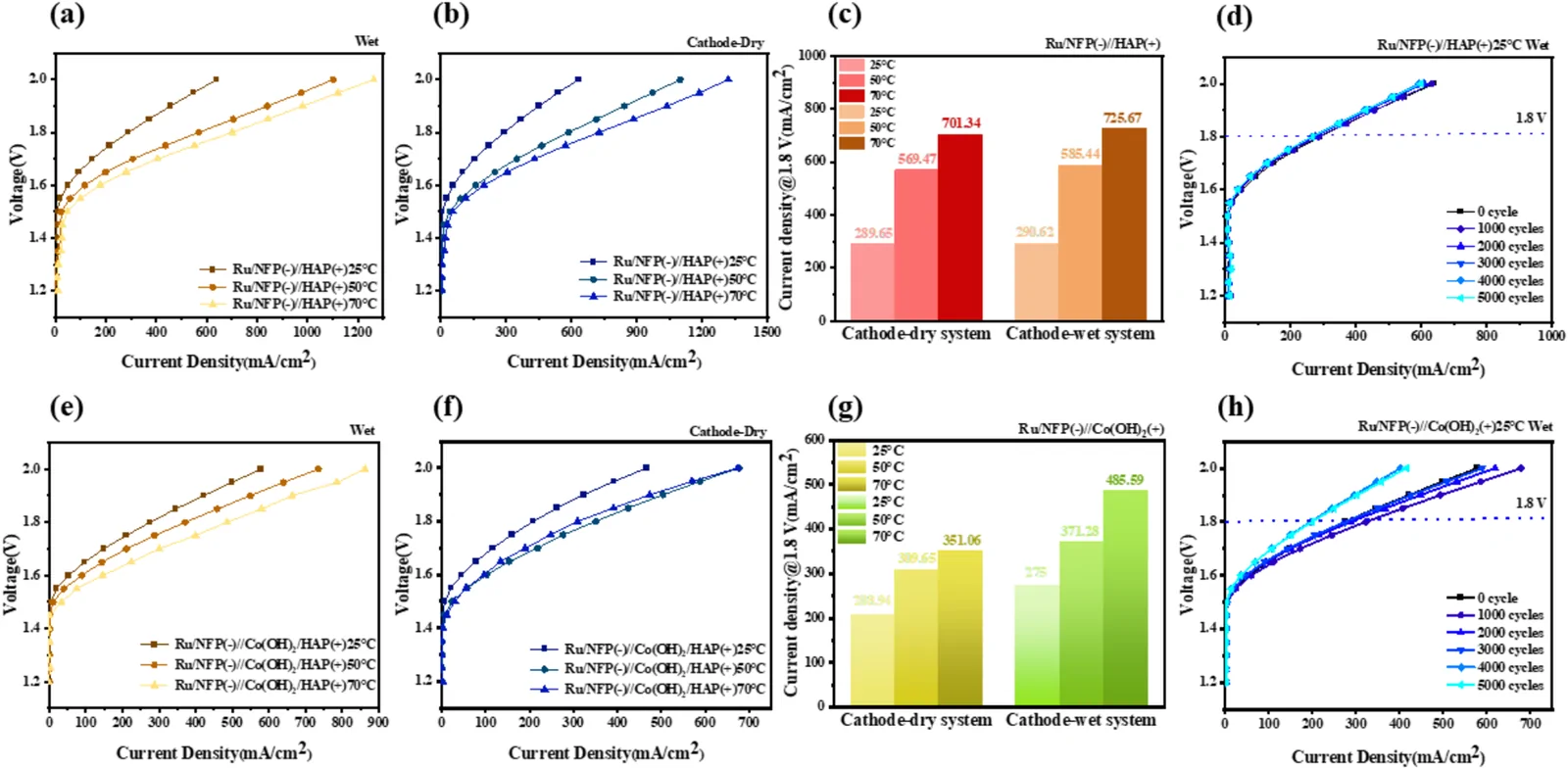
Single-cell AEMWE performance and short-term stability evaluation: polarization (*I*–*V*) curves of the Ru/NFP(−) // HAP­(+) system under (a) wet and (b) cathode-dry conditions at 25, 50, and 70 °C. (c) Corresponding current density at 1.8 V. (d) Accelerated stress test (AST) evaluating stability over 5000 cycles at 25 °C. Polarization (*I*–*V*) curves of the Ru/NFP(−) // Co­(OH)_2_/HAP­(+) system under (e) wet and (f) cathode-dry conditions at 25, 50, and 70 °C. (g) Corresponding current density at 1.8 V. (h) Stability evaluation over 5000 cycles at 25 °C.

To further evaluate system stability, accelerated stress tests (AST) were conducted at 25 °C with the cathode maintained under wet conditions. During the test, the current density was alternated in a square-wave manner between 500 mA cm^–2^ and 100 mA cm^–2^ at 5 s intervals for a total of 5000 cycles, corresponding to an overall test duration of approximately 13.9 h, as shown in Figure 9(d,h). The results indicate that the Co­(OH)_2_/HAP material system exhibited more significant voltage degradation after 5000 AST.^64^ Particularly, the performance of the Co­(OH)_2_/HAP material system improves first and then degrades later in the 5000-cycle AST. This suggests strong surface activation and reconstruction of the Co­(OH)_2_ catalyst layer.

Although material characterization shows that Co­(OH)_2_ modification transforms the catalyst from Co­(OH)_2_ to CoOOH, theoretically providing enhanced electrochemical activity and optimized electronic structure, such as a higher proportion of active oxidation states and lower charge-transfer resistance, the single-cell test results (Figure 9(e,f,g)) reveal that the overall performance of the Ru/NFP(−)//Co­(OH)_2_/HAP­(+) system is still lower than that of the unmodified Ru/NFP(−)//HAP­(+) system. Specifically, under wet cathode conditions at 70 °C, the Ru/NFP(−)//HAP­(+) system reaches 725.67 mA cm^–2^ at 1.8 V, whereas the Ru/NFP(−)//Co­(OH)_2_/HAP­(+) system only achieves 485.59 mA cm^–2^.

These results indicate that improvements in intrinsic catalytic activity do not necessarily translate directly to single-cell performance, which may be limited by mass-transport constraints within the catalyst layer and porous transport structure.^65^

Based on the measured mass loading of Co­(OH)_2_ (1.2 mg cm^–2^) over a 3 × 3 cm^2^ deposition area, an equivalent planar thickness on the order of a few micrometers can be estimated. As observed in the SEM images, the electrodeposited Co­(OH)_2_ exhibits a nanosheet-like morphology distributed over the fibrous HAP network. While SEM provides qualitative morphological information, these observations suggest that the deposited Co­(OH)_2_ partially covers the fiber surfaces and may extend into the interfiber voids rather than forming a uniform continuous film. At this loading level, such structural features may reduce effective pore accessibility and hinder oxygen bubble release within the porous electrode, particularly under high-current density operation. These effects may therefore contribute to the mass-transport limitations observed in single-cell performance.

The literature reports that the morphology and thickness of the catalyst layer, as well as its integration with the ion conductor and porous transport layer (PTL), significantly affect catalyst utilization and the transport of gases, hydroxide ions, and reactants.^65,66^ In AEMWE systems, such effects may even be more critical than the intrinsic catalytic activity. Since the same Ru/NFP cathode was employed for both the Ru/NFP(−)//HAP­(+) and Ru/NFP(−)//Co­(OH)_2_/HAP­(+) configurations, the cathodic contribution is expected to remain comparable throughout the single-cell evaluation. Therefore, the performance differences observed between the two systems are interpreted primarily in terms of the different anode structures and their associated transport characteristics.

As shown in Figure 9(e–h), even though Co­(OH)_2_ modification enhances the material-level activity, its practical performance in the cell may still be limited by transport effects. SEM observations indicate that Co­(OH)_2_ deposition alters the surface morphology of the HAP fibers. Such morphological changes may influence electrolyte accessibility and gas transport within the porous electrode, which could contribute to the observed decrease in single-cell performance at high current densities.^67^ Furthermore, the increased surface coverage associated with Co­(OH)_2_ deposition may hinder reactant accessibility and gas transport within the porous electrode, particularly under high-current density conditions.

Therefore, single-cell performance depends not only on the intrinsic activity of the catalyst but also on electrode–membrane interface contact, two-phase flow behavior, and mass-transport properties in the PTL.^65,67^

The AST results and the 60 h galvanostatic stability test provide useful information regarding the short-term operational stability and degradation trends. However, these measurements are not sufficient to establish the long-term durability under practical AEMWE operating conditions. Extended-duration testing will be required to further evaluate degradation mechanisms associated with catalyst stability, membrane durability, electrolyte evolution, and transport-related losses during the prolonged operation. In summary, although Co­(OH)_2_ modification improves the electrochemical properties at the material level, the single-cell results suggest that transport-related effects remain important under practical AEMWE operating conditions. Further optimization of the electrode architecture and mass-transport pathways may be beneficial for improving device-level performance.

## Conclusions

This study demonstrates the significant potential of the HAP-based asymmetric electrode configuration for practical AEMWE applications. The Ru/NFP(−)//HAP­(+) system maintains electrolysis performance under both cathode-wet and cathode-dry configurations, highlighting its strong practical feasibility. Although Co­(OH)_2_ modification slightly improves the electronic structure and intrinsic catalytic activity at the material level, this advantage does not fully translate to enhanced single-cell performance, emphasizing the critical role of mass-transport resistance during cell integration. Overall, these findings highlight the importance of balancing intrinsic catalytic activity with optimized mass transport in the AEMWE electrode design and demonstrate the potential of HAP as a promising PTL substrate for AEMWE applications within the investigated operating window. The observed performance degradation during durability testing further suggests that transport-related losses and interfacial evolution may become increasingly important during the prolonged operation. Further long-duration testing is required to evaluate the durability under practical operating conditions.

## Supplementary Material



## Notes

SEM micrographs of (a) NFP, (b) Ru/NFP, (c) AST-treated Ru/NFP, and (d) LT-treated Ru/NFP at magnifications of 100×, 1,000×, and 10,000× (Figure S1); EDS mapping of HAP (Figure S2); EDS mapping of Co­(OH)_2_/HAP (Figure S3); EDS mapping of AST-Co­(OH)_2_/HAP (Figure S4); EDS mapping of LT-Co­(OH)_2_/HAP (Figure S5); EDS mapping of NFP (Figure S6); EDS mapping of Ru/NFP (Figure S7); EDS mapping of AST-Ru/NFP (Figure S8); EDS mapping of LT-Ru/NFP (Figure S9); EDS spectra of (a) HAP, (b) Co­(OH)_2_/HAP, (c) AST-Co/HAP, and (d) LT-Co/HAP (Figure S10); EDS spectra of (a) NFP, (b) Ru/NFP, (c) AST- Ru/NFP, and (d) LT- Ru/NFP (Figure S11); XRD patterns of NFP, Ru/NFP, AST- Ru/NFP, and LT- Ru/NFP (Figure S12); XPS survey spectra of (a) NFP, (b) Ru/NFP, (c) AST- Ru/NFP, and (d) LT- Ru/NFP (Figure S13) (PDF)
