# Supplementary material for: Self-Catalyzed Hastelloy Fiber Paper (HAP) Porous Transport Layer for Oxygen Evolution Reaction in Anion Exchange Membrane Water Electrolysis
Source: Langmuir. 2026 Oct 8;42(41):30912–23. doi: 10.1021/acs.langmuir.6c04347 (PMC13689907; doi:10.1021/acs.langmuir.6c04347)
Supplement: Supplementary file 1 [file la6c04347_si_001.pdf]

Supporting Information

Self-catalyzed Hastelloy Fiber Paper (HAP) Porous Transport Layer for Oxygen Evolution Reaction in Anion Exchange Membrane Water Electrolysis

Yu-Chen Chang[1], Shuo-En Yu[2] , I-Chun Cheng[3], Jian-Zhang Chen [1,4,5,6,]*

[1] Graduate School of Advanced Technology, National Taiwan University, Taipei City 106319, Taiwan

[2] Institute for Solid State Physics, The University of Tokyo, Chiba 277-8581, Japan

[3] Graduate Institute of Photonics and Optoelectronics and Department of Electrical Engineering, National Taiwan University, Taipei City 106319, Taiwan

[4] Institute of Applied Mechanics, National Taiwan University, Taipei City 106319, Taiwan

[5] Advanced Research Center for Green Materials Science and Technology, National Taiwan University, Taipei City 106319, Taiwan

[6] Research Center for Applied Sciences, Academia Sinica, Taipei City 115201, Taiwan

** Correspondence: jchen@ntu.edu.tw; Tel.: +886-2-3366-5694*

List:

Figure S1. SEM micrographs of (a) NFP, (b) Ru/NFP, (c) AST-treated Ru/NFP, and (d) LT-treated Ru/NFP at magnifications of 100×, 1,000×, and 10,000×.

Figure S2. EDS mapping of HAP.

Figure S3. EDS mapping of $Co(OH)_2$/HAP.

Figure S4. EDS mapping of AST-$Co(OH)_2$/HAP.

Figure S5. EDS mapping of LT-$Co(OH)_2$/HAP.

Figure S6. EDS mapping of NFP.

Figure S7. EDS mapping of Ru/NFP.

Figure S8. EDS mapping of AST-Ru/NFP.

Figure S9. EDS mapping of LT-Ru/NFP.

Figure S10. EDS spectra of (a) HAP, (b) $Co(OH)_2$/HAP, (c) AST-$Co(OH)_2$/HAP, and (d) LT-$Co(OH)_2$/HAP.

Figure S11. EDS spectra of (a) NFP, (b) Ru/NFP, (c) AST- Ru/NFP, and (d) LT-Ru/NFP.

Figure S12. XRD patterns of NFP, Ru/NFP, AST- Ru/NFP, and LT- Ru/NFP.

Figure S13. XPS survey spectra of (a) NFP, (b) Ru/NFP, (c) AST- Ru/NFP, and (d) LT- Ru/NFP.

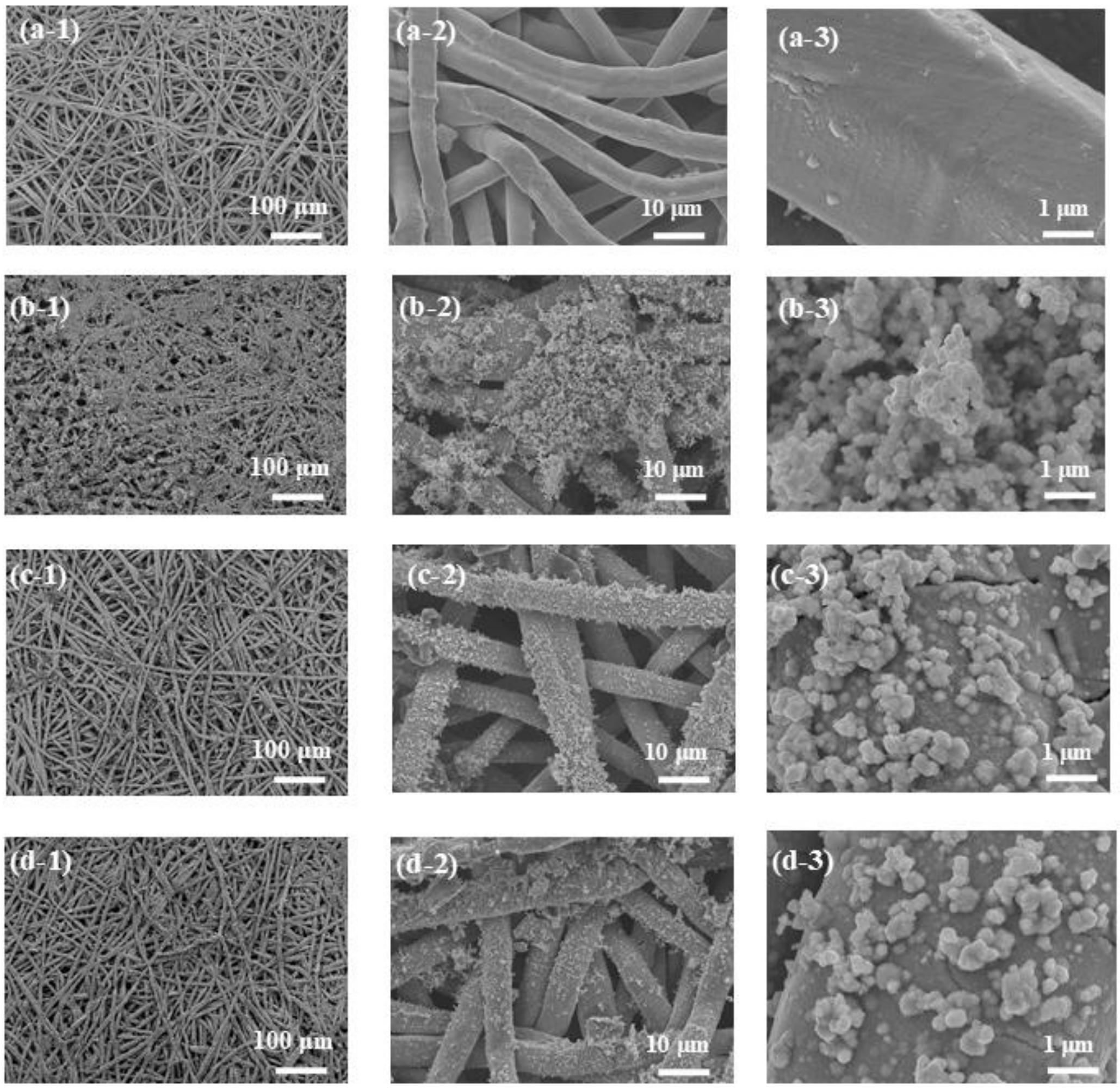

Figure S1. SEM micrographs of (a) NFP, (b) Ru/NFP, (c) AST-treated Ru/NFP, and (d) LT-treated Ru/NFP at magnifications of 100×, 1,000×, and 10,000×.

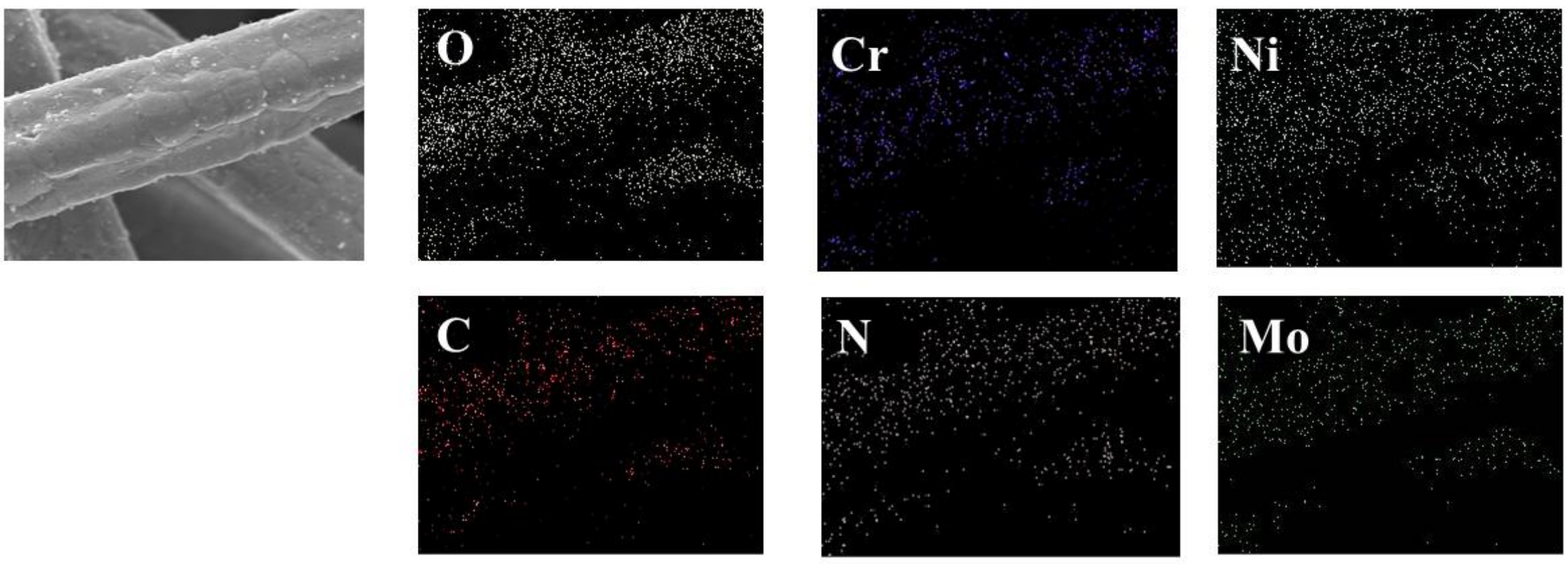

Figure S2. EDS mapping of HAP.

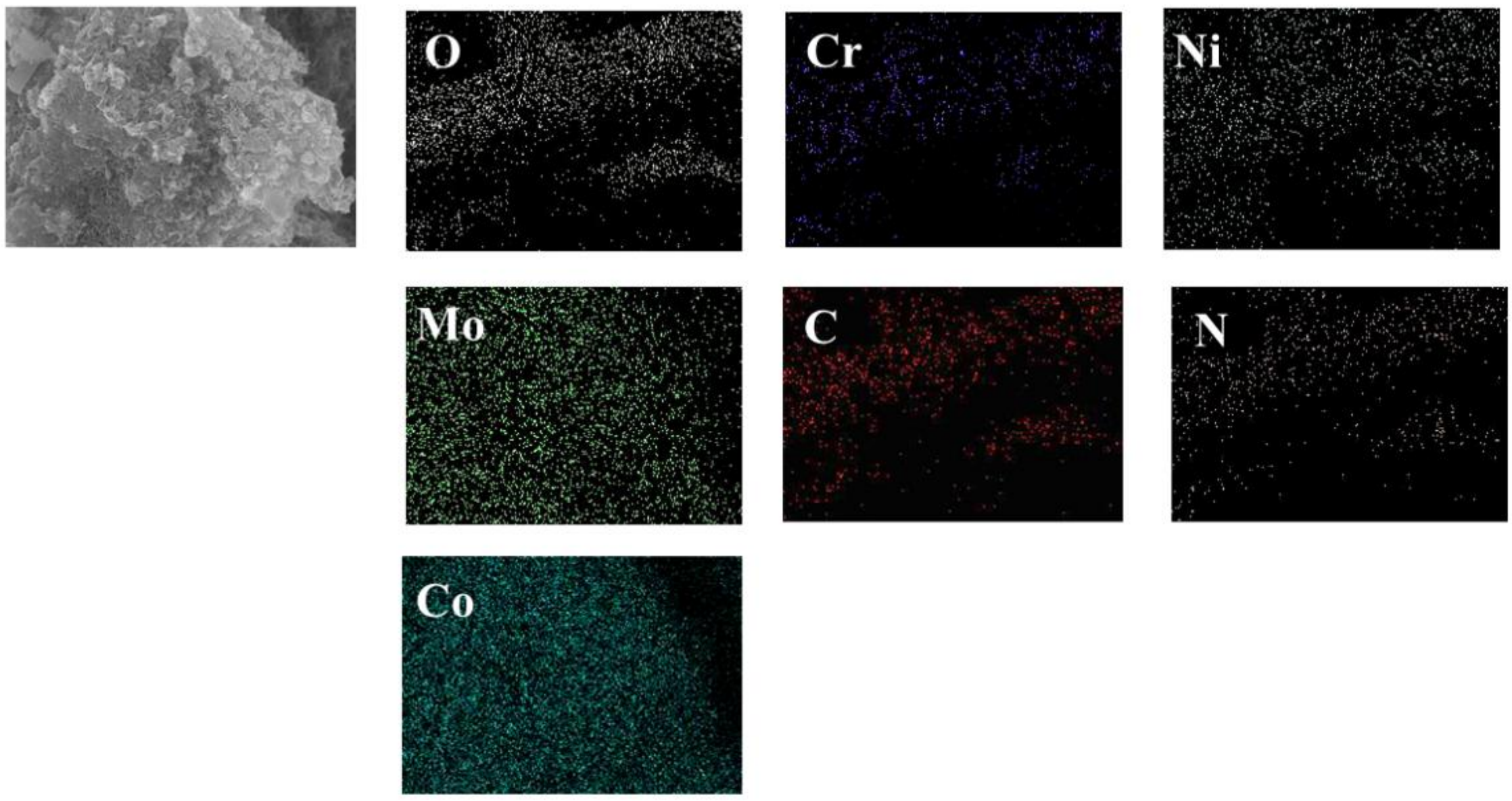

Figure S3. EDS mapping of $Co(OH)_2$/HAP.

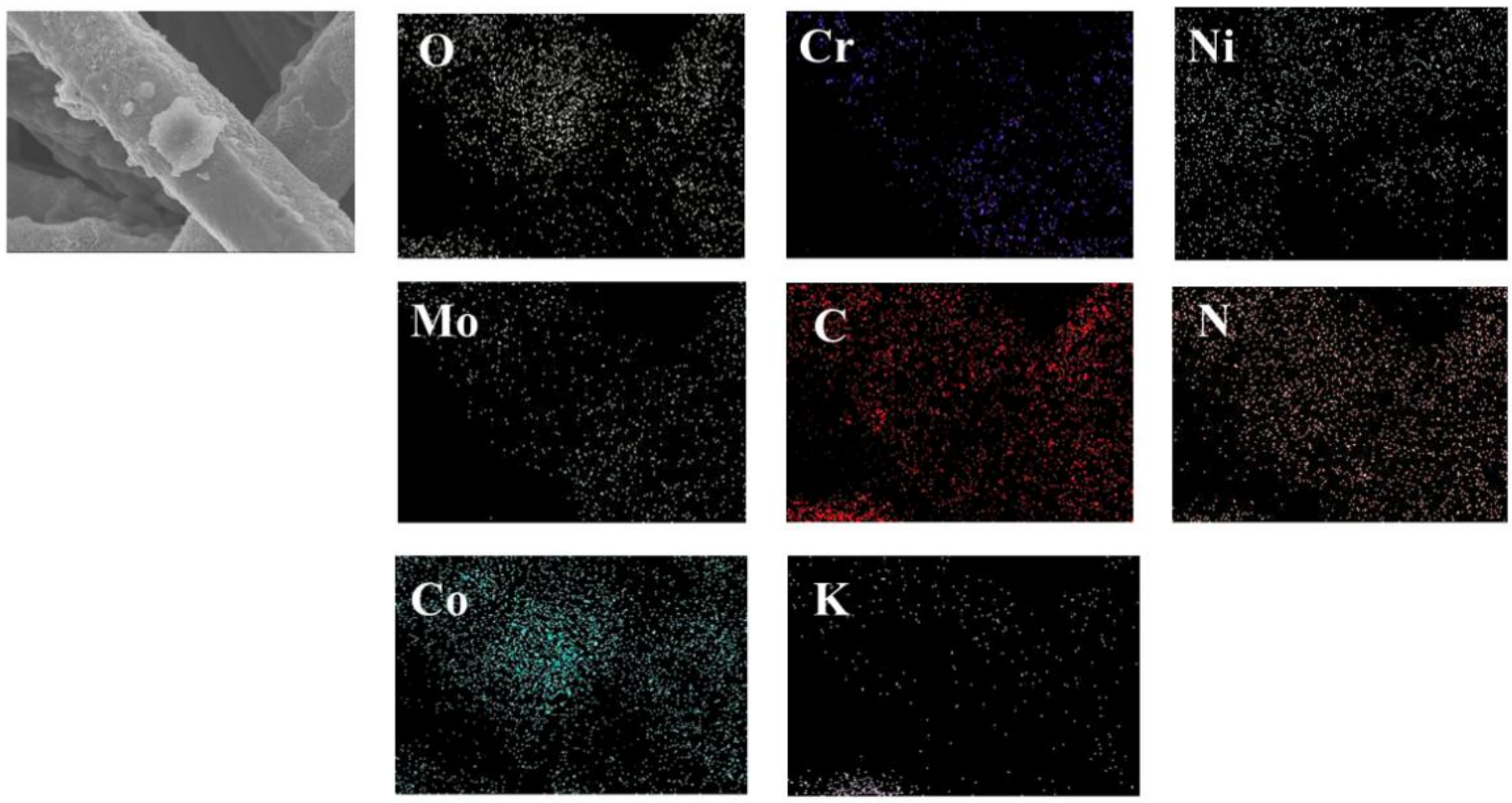

Figure S4. EDS mapping of AST-$Co(OH)_2$/HAP.

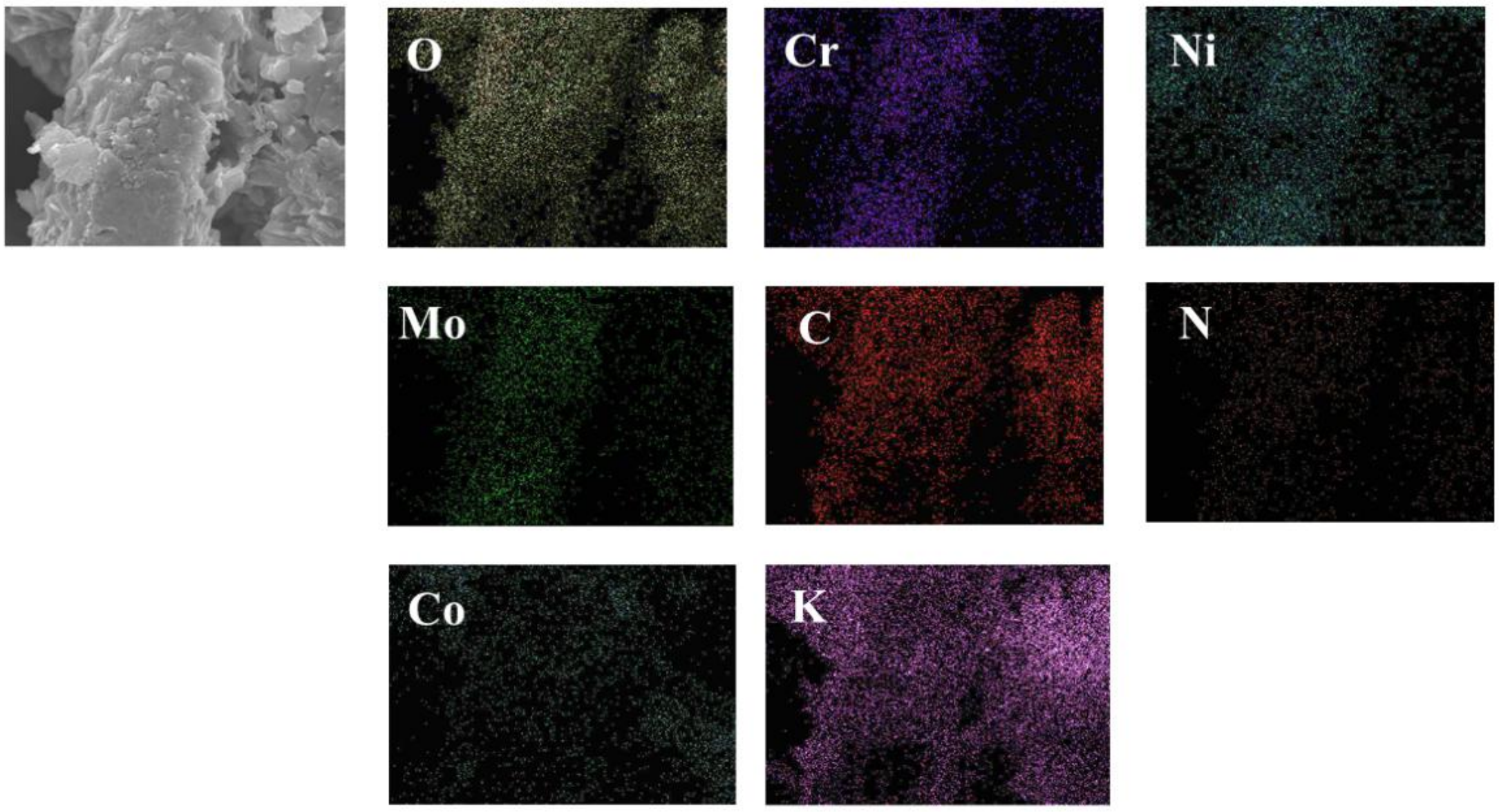

Figure S5. EDS mapping of LT-$Co(OH)_2$/HAP.

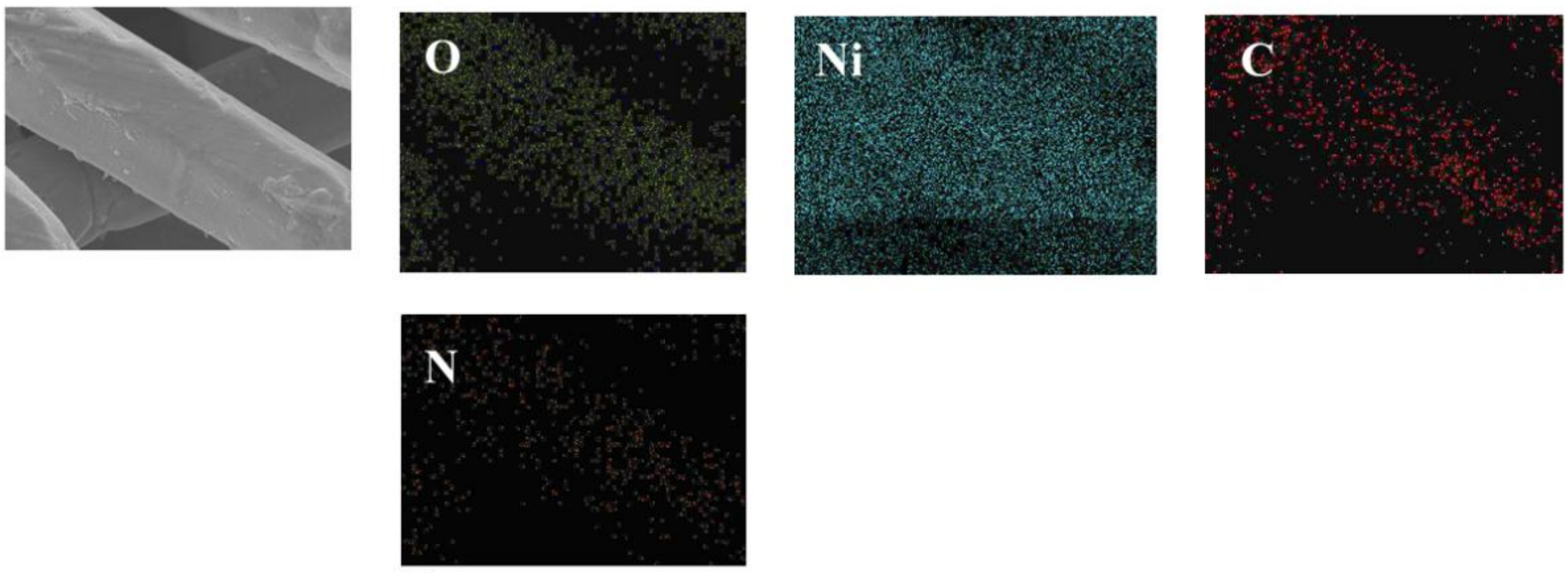

Figure S6. EDS mapping of NFP.

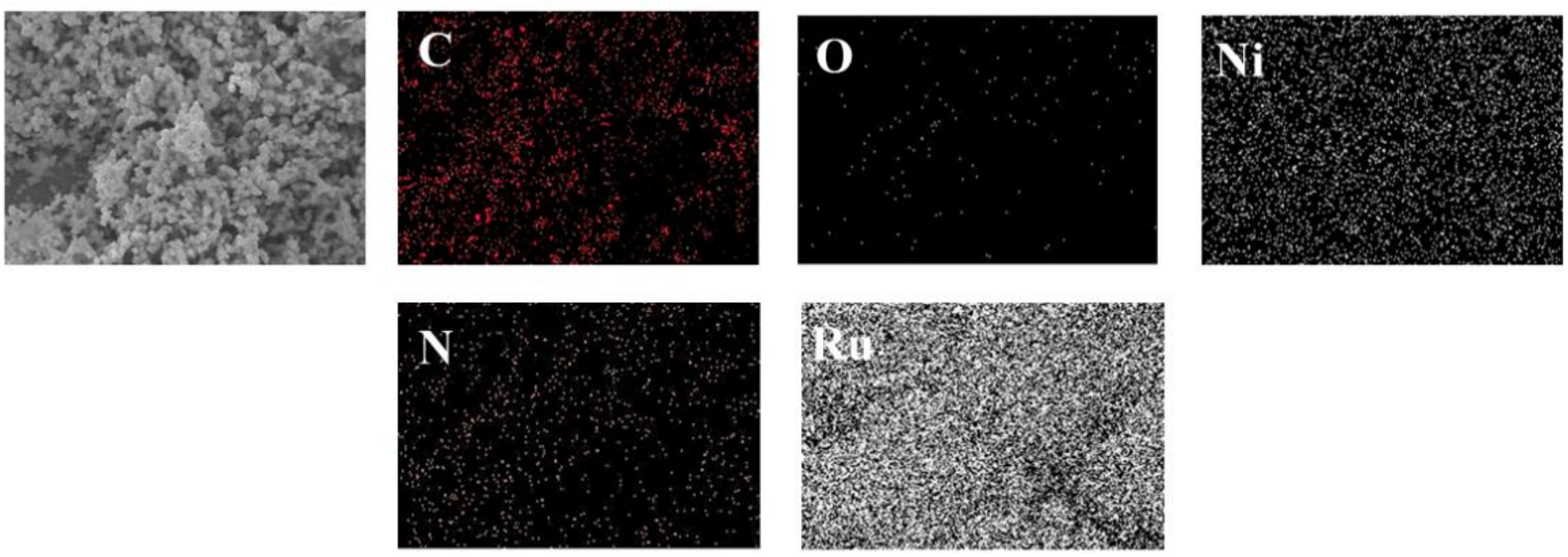

Figure S7. EDS mapping of Ru/NFP.

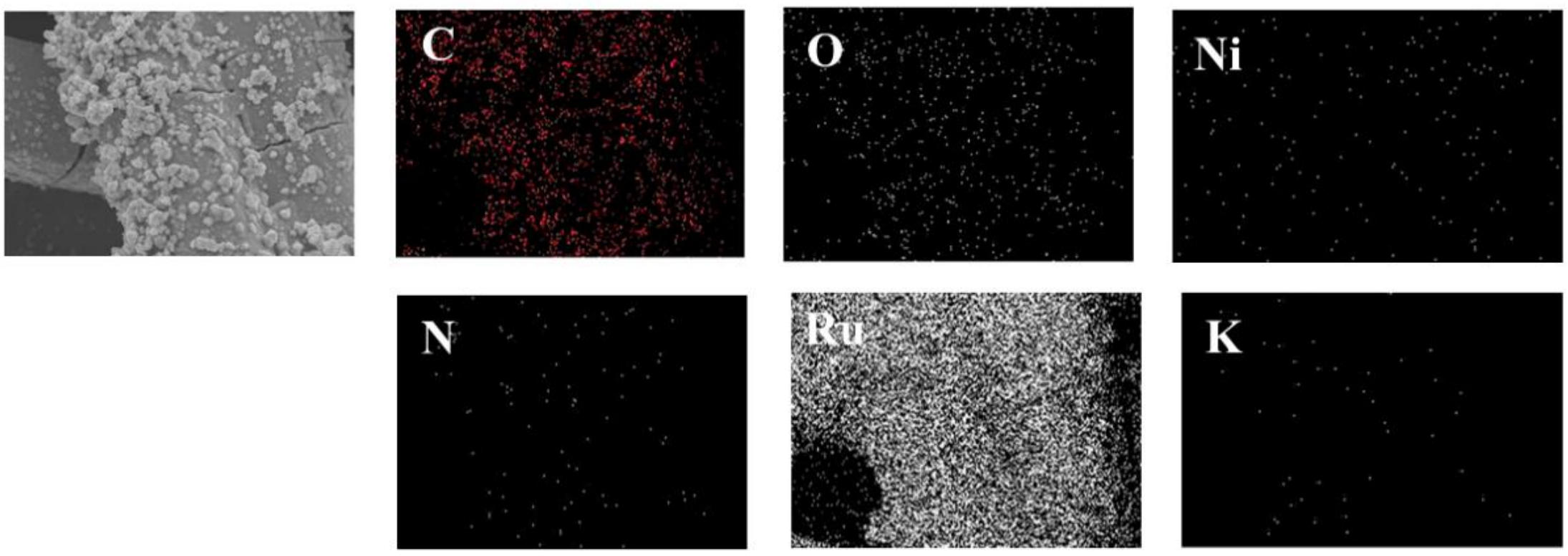

Figure S8. EDS mapping of AST-Ru/NFP.

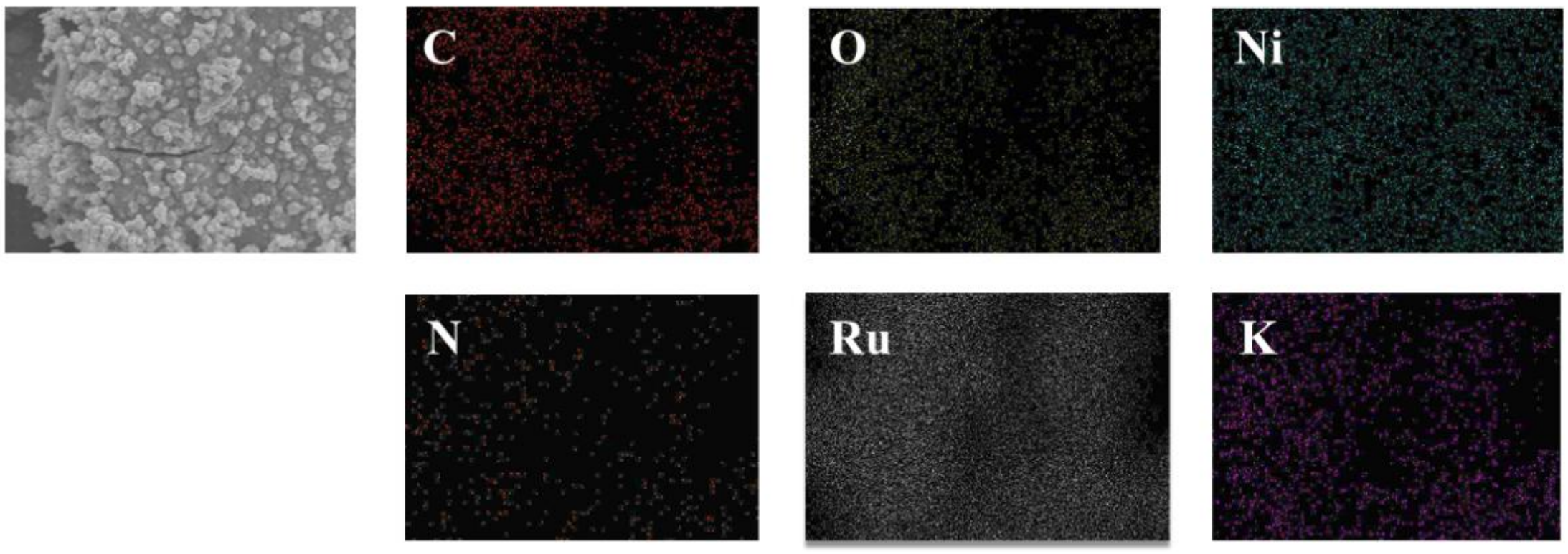

Figure S9. EDS mapping of LT-Ru/NFP.

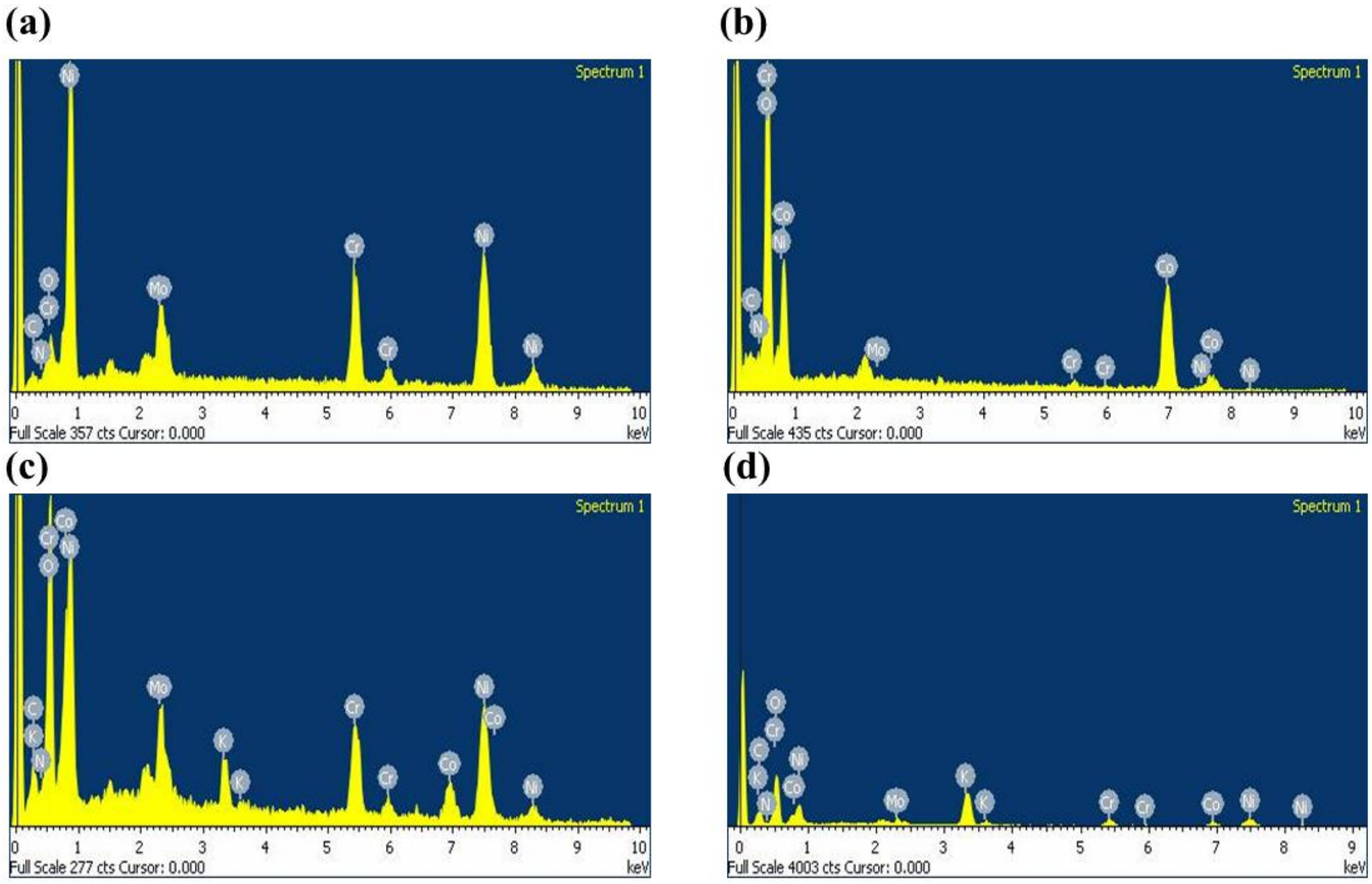

Figure S10. EDS spectra of (a) HAP, (b) $Co(OH)_2$/HAP, (c) AST-$Co(OH)_2$/HAP, and (d) LT-$Co(OH)_2$/HAP.

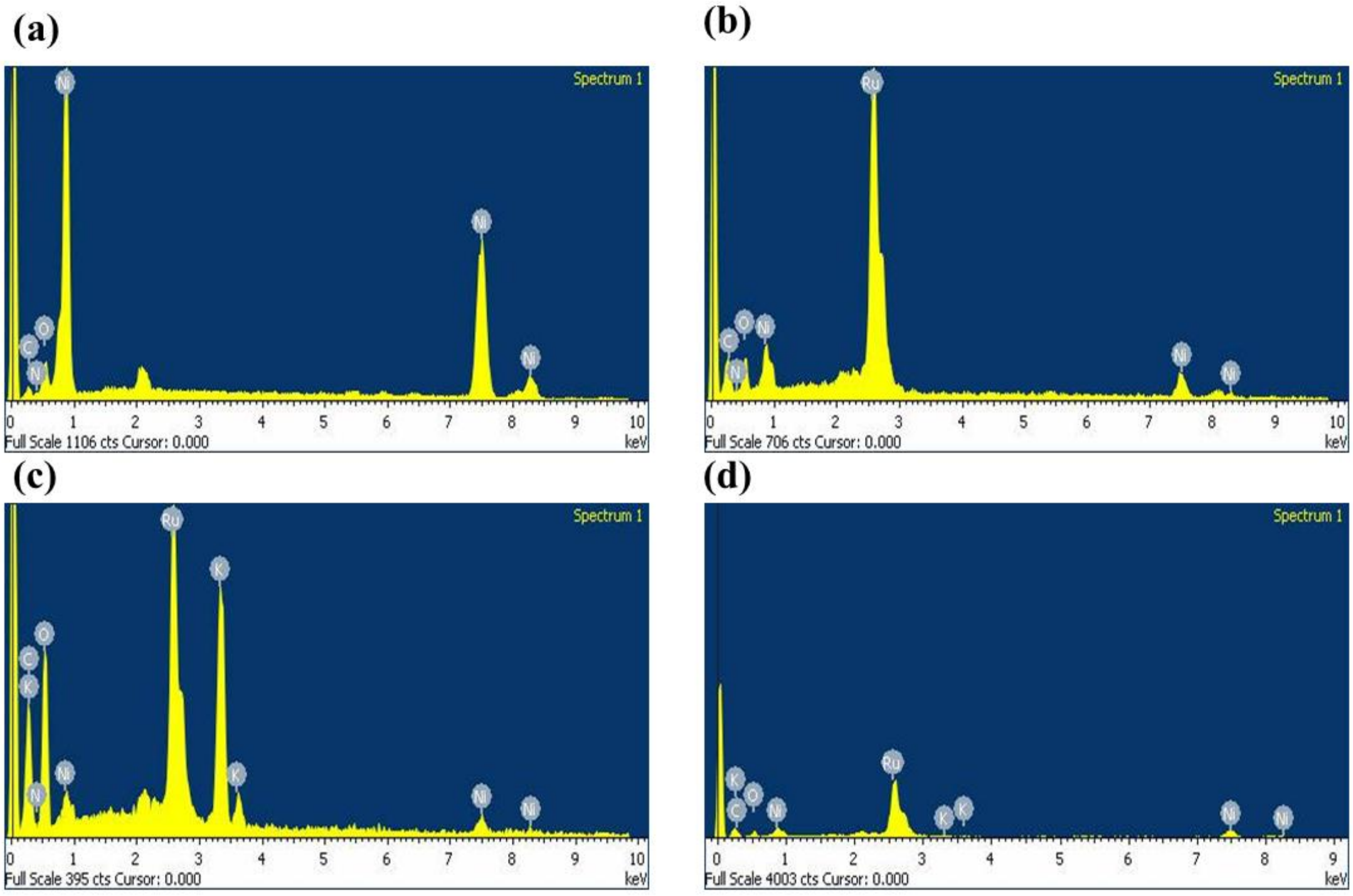

Figure S11. EDS spectra of (a) NFP, (b) Ru/NFP, (c) AST- Ru/NFP, and (d) LT-Ru/NFP.

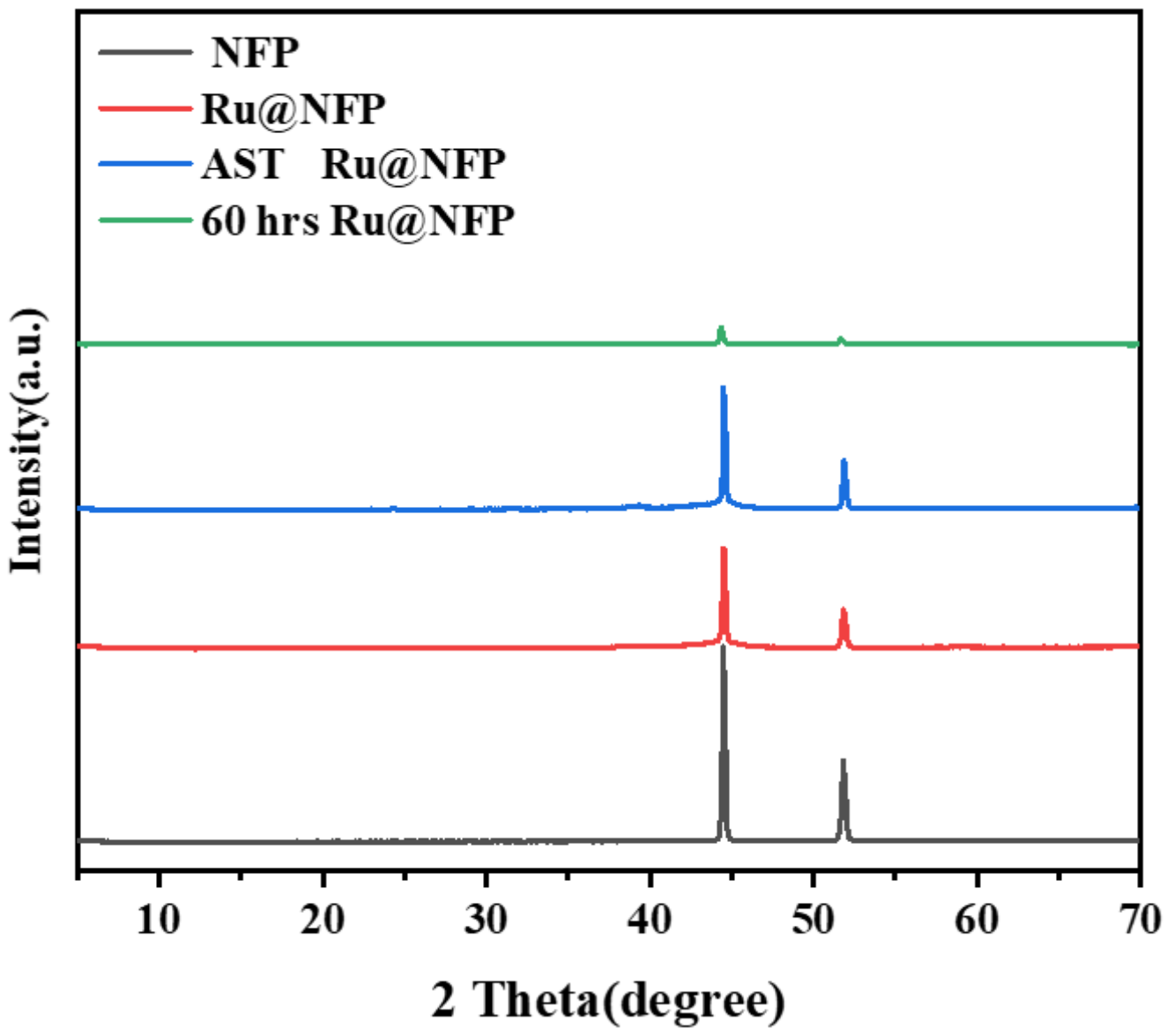

Figure S12. XRD patterns of NFP, Ru/NFP, AST- Ru/NFP, and LT- Ru/NFP.

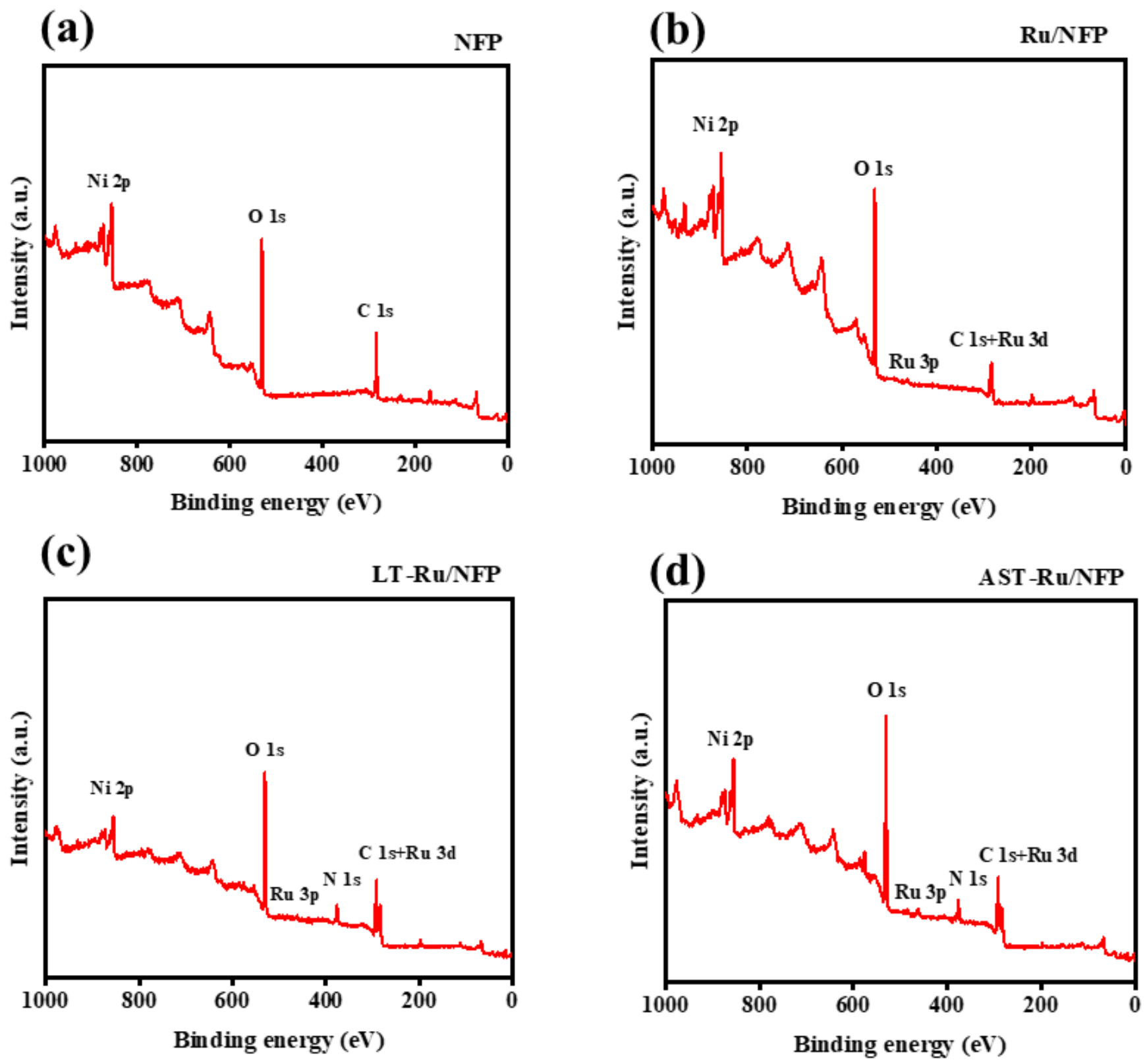

Figure S13. XPS survey spectra of (a) NFP, (b) Ru/NFP, (c) AST- Ru/NFP, and (d) LT- Ru/NFP.
